# Genotyping and biofilm formation of *Mycoplasma hyopneumoniae* and their association with virulence

**DOI:** 10.1186/s13567-022-01109-x

**Published:** 2022-11-17

**Authors:** Yuzi Wu, Yanfei Yu, Lizhong Hua, Yanna Wei, Yuan Gan, Hafizah Yousuf Chenia, Yixuan Wang, Xing Xie, Jia Wang, Maojun Liu, Guoqing Shao, Qiyan Xiong, Zhixin Feng

**Affiliations:** 1 Institute of Veterinary Medicine, Jiangsu Academy of Agricultural Sciences, Key Laboratory of Veterinary Biological Engineering and Technology, Ministry of Agriculture and Rural Affairs, Nanjing, China; 2 School of Food and Biological Engineering, Jiangsu University, Zhenjiang, China; 3 College of Veterinary Medicine, Nanjing Agricultural University, Nanjing, China; 4 Discipline of Microbiology, School of Life Sciences, University of KwaZulu-Natal, Durban, South Africa; 5 University of Warwick, Coventry, CV4 7AL UK; 6 School of Life Sciences, Jiangsu University, Zhenjiang, China; 7 School of Animal Husbandry and Veterinary Medicine, Jiangsu Vocational College of Agriculture and Forestry, Jurong, China

**Keywords:** *Mycoplasma hyopneumoniae*, virulence, genotyping, biofilm

## Abstract

*Mycoplasma hyopneumoniae*, the causative agent of swine respiratory disease, demonstrates differences in virulence. However, factors associated with this variation remain unknown. We herein evaluated the association between differences in virulence and genotypes as well as phenotype (i.e., biofilm formation ability). Strains 168 L, RM48, XLW-2, and J show low virulence and strains 232, 7448, 7422, 168, NJ, and LH show high virulence, as determined through animal challenge experiments, complemented with in vitro tracheal mucosa infection tests. These 10 strains with known virulence were then subjected to classification via multilocus sequence typing (MLST) with three housekeeping genes, P146-based genotyping, and multilocus variable-number tandem-repeat analysis (MLVA) of 13 loci. MLST and P146-based genotyping identified 168, 168 L, NJ, and RM48 as the same type and clustered them in a single branch. MLVA assigned a different sequence type to each strain. Simpson’s index of diversity indicates a higher discriminatory ability for MLVA. However, no statistically significant correlation was found between genotypes and virulence. Furthermore, we investigated the correlation between virulence and biofilm formation ability. The strains showing high virulence demonstrate strong biofilm formation ability, while attenuated strains show low biofilm formation ability. Pearson correlation analysis revealed a significant positive correlation between biofilm formation ability and virulence. To conclude, there was no association between virulence and our genotyping data, but virulence was found to be significantly associated with the biofilm formation ability of *M. hyopneumoniae*.

**Supplementary Information:**

The online version contains supplementary material available at 10.1186/s13567-022-01109-x.

## Introduction

The incidence of mycoplasma pneumonia of swine (MPS) has witnessed an increase, resulting in significant economic losses to the pig industry across the world [1]. The pathogenic effects of different *Mycoplasma hyopneumoniae* strains on pigs are variable. However, little remains known about the reason or elements responsible for such differences. In this study, our aim was to identify factors associated with variations in *M. hyopneumoniae* virulence.

Animal challenge experiments [2], host cell adhesion [3], genotyping [4], and biofilm formation ability [5] have been previously used to assess virulence and pathogenicity of *M. hyopneumoniae* strains. Pig challenge experiments are the gold standard method for virulence assessment. The virulence characteristics of *M. hyopneumoniae* strains can be evaluated using in vitro models, such as swine tracheal mucosa. Some studies have explored the association between *M. hyopneumoniae* virulence and genotypes. Genotyping plays a vital role in epidemiological investigations and in bacterial identification and differentiation. Genotyping techniques are usually easy to perform, require minimal bacterial load, and results are quickly generated [4]. Multilocus sequence typing (MLST), P146-based genotyping, and multilocus variable-number tandem-repeat (VNTR) analysis (MLVA) have become increasingly popular for the molecular typing of bacteria [6], considering that they can validate predominant genotypes, analyze evolutionary relationships, and reveal variations in dominant types [7]. The application of these methods, particularly MLVA, has been reported to assess the virulence of *M. hyopneumoniae* strains. MLVA typing of *M. hyopneumoniae* indicate an association between the number of tandem amino acid repeats in P97 and other adhesins, including P76, P146, and P216, and virulence [8, 9]. Minion et al. found that cilium adherence was not significant until there were at least eight repeats of AAKPV/E in P97 R1 [10]. In contrast, Stakenborg et al. found that a pathogenic strain contained only two repeats of AAKPV/E in P97 R1 [11]. Another study showed that no MLVA clusters were associated with *M. hyopneumoniae* virulence [12]. As shown from these contradictory findings, the association between genotypes and virulence of *M. hyopneumoniae* remains controversial.

Some studies have suggested the presence of a relationship between biofilm formation ability and bacterial virulence [13]. Biofilms are dense surface-associated bacterial communities, and they protect bacteria from the lethal effects of host immunity and antibacterial agents [14]. Biofilm formation is a risk factor for mortality in some cases, and the ability to form biofilms is now widely accepted as one of the main pathogenesis-related virulence factors [15]. Inhibiting the expression of virulence-associated genes is reportedly correlated with the attenuation of the biofilm formation ability of *Stenotrophomonas maltophilia* [16]. Further, the biofilm formation ability of *Histophilus somni* seems to facilitate its persistence in systemic sites [17]. Another study indicated that decreased expression of virulence-related genes inhibits the ability of *Staphylococcus aureus* to form biofilms [18]. Nevertheless, different results have been reported. The decreased ability of *Plesiomonas shigelloides* to infect Caco-2 cells is accompanied by an enhanced ability to form biofilms [19]. Furthermore, a virulent strain of *Streptococcus suis* was found to lower its virulence by forming biofilms so as to establish a persistent infection in vivo [20]. However, little has been reported about the relationship between biofilms and differences in virulence among *M. hyopneumoniae* strains.

Herein we first performed pig challenge experiments to assess the virulence characteristics of *M. hyopneumoniae* strains. The results were further complemented and re-evaluated in parallel by performing in vitro tracheal mucosa infection tests. The association among virulence, genotypes and biofilm formation ability of *M. hyopneumoniae* was subsequently evaluated. Genotypes were assessed by MLST, P146-based genotyping, and MLVA, followed by phylogenetic analyses. The biofilm formation ability of *M. hyopneumoniae* strains was assessed with the microtiter plate biofilm assay (OD_570_). Finally, the relationship between the virulence characteristics of *M. hyopneumoniae* and genotypes as well as the correlation between biofilms and virulence were investigated.

## Materials and methods

### Characterization of *M. hyopneumoniae* virulence

#### *M. hyopneumoniae* strains

*M. hyopneumoniae* strain J, which is non-pathogenic, was purchased from American Type Culture Collection [21]. The commercialized vaccine strains 168 L and RM48 (an attenuated live vaccine strain) were purchased from JOFUNHWA Biotechnology Corporation, Nanjing, China and Zhejiang CEVA EBVAC Biotech Corporation, Hangzhou, China, respectively. Four field strains, namely 168, NJ, XLW-2, and LH, were isolated from pig lung tissues during MPS outbreaks. Strain 168 was isolated from Gansu Province, China in 1974 [22], and NJ, XLW-2 and LH were isolated from Jiangsu Province, China in 2004, 2010 and 2016 respectively. Strain 168 L was derived from strain 168 by > 300 continuous passages in vitro [3]. Strains 232 [23], 7448 [24], and 7422 [25] are recognized as pathogenic strains worldwide.

#### Comparing virulence of field strains by animal challenge experiments

Animal challenge experiments were performed to evaluate and compare the virulence of strains 168 F115, 168 L F335, NJ F41, XLW-2 F22, and LH F10. Sera samples from 18 Bama miniature pigs were tested for *M. hyopneumoniae* infection using a commercial antibody test kit (IDEXX HerdChek® *Mycoplasma hyopneumoniae* Antibody ELISA Test Kit, IDEXX Laboratories Inc., Westbrook, ME, USA). The animals were equally distributed in six different groups. Pigs from different groups were housed in individual pens with metal slatted flooring and metal gated sides and given water and a commercial antibiotic-free pelleted diet. For challenge via the tracheal route, 5 mL of 5 × 10^8^ color changing units/mL of *M. hyopneumoniae* suspension was used. Each pig in the negative-control (NC) group was administered 5 mL sterile KM2 medium. The groups challenged with different strains and the NC group were kept in separate rooms with independent ventilating systems. After challenge, pigs were monitored in the morning daily by the same person for clinical signs, including apathy, loss of appetite, coughing or wheezing. The animals were euthanized 28 days post-infection. After necropsy, pathological changes in various parts of the lungs were separately scored. A lung lesion score was derived using a previous method, with some modifications, to make score distinction more evident [26]: in the absence of any lesions, the lobes were rated as 0 points, 0.1–12.5% as 0.5 points, 12.6–25% as 1 point, 25.1–37.5% as 1.5 points, 37.6–50% as 2 points, 50.1–62.5% as 2.5 points, 62.6–75% as 3 points, 75.1–87.5% as 3.5 points, and 87.6–100% as 4 points. The lung pathological change index was then calculated (up to 28 points) for each pig. In addition, hematoxylin and eosin (HE) staining was performed to assess pathological changes in the lungs. Experiments were performed as previously described with a minor modification [27]. From each pig, DNA was extracted from 1 mg lung tissues taken from the margin between healthy and diseased tissues and qPCR was performed for the quantitative analysis of *M. hyopneumoniae* [28]. The virulence of strains was analyzed as follows, high virulence isolates significantly differed from the negative control group. Low virulence isolates did not show significant differences from the negative control group, but they were significantly different from high virulence isolates in terms of the lung lesion score [29]. Macroscopic lesions, clinical signs, and HE staining data were assessed for verification. All experimental procedures were approved by the Ethical and Animal Welfare Committee of the Jiangsu Academy of Agricultural Sciences, China [SYXK (Su) 2015–0019].

#### Assessing the ability of the strains to infect the tracheal mucosa

The tracheas were aseptically excised from healthy pigs and submerged in chilled Hanks balanced salt solution (HBSS, 14170112, Gibco, USA). The cartilages were removed from the outside edge of the tracheal ring for easier imaging. The processed tracheas were cut into pieces (1 × 1 cm), rinsed thrice with HBSS, and then incubated with RPMI-1640 (31870082, Gibco, USA) overnight at 37 °C and 5% CO_2_ to ensure they were sterile [30]. Subsequently, J F14, 168 F115, 168 L F335, RM48 F792, NJ F41, XLW-2 F22, and LH F10 (10^8^ CCU, 1 mL) were incubated with the tracheal mucosa separately in a 24-well plate for 24 h at 37 °C and 5% CO_2_. The one incubated with RPMI-1640 media (i.e., uninfected) served as the NC. After incubation, the tracheal mucosa was rinsed thrice with HBSS to wash away planktonic bacteria. The experiments were performed in triplicate. Each tracheal mucosa was cut into two equal parts. One part was fixed in 2.5% glutaraldehyde and examined under a Zeiss EVO-LS10 scanning electron microscope (Zeiss, Germany) to evaluate micromorphic changes on the surface of ciliary epithelial cells, while the other part was used for live/dead cell counting to assess cell viability. The LIVE/DEAD^®^ BacLight^™^ Bacterial Viability Kit (Thermo Fisher Scientific Inc., USA) was used for cell fluorescence staining, according to the manufacturer instructions. The prepared specimens were then observed under an UltraVIEW VoX laser scanning confocal microscope (PerkinElmer, USA). Cell viability is presented as the mean fluorescence intensity (MFI) ratio of red to green (R/G ratio), which represents the ratio of dead to live mucosal epithelial cells. To further evaluate if there was a statistically significant correlation between virulence and in vitro tracheal mucosa infection test results, non-conditional logistic regression analysis was performed using the IBM SPSS Statistics software (v 18.0). If the *p* value of lung lesion score or R/G ratio calculated by the single sample K-S test was > 0.05, then lung lesion score or R/G ratio was regarded to be normally distributed and could be analyzed by Pearson correlation analysis. Pearson correlation coefficient (r) of lung lesion score and R/G ratio > 0.8 and *p* value < 0.01 indicated an extremely strong correlation [31].

### Genotyping of *M. hyopneumoniae* strains

#### Genotyping methods

Seven different *M. hyopneumoniae* strains, including J F14, 168 F115, 168 L F335, RM48 F792, NJ F41, XLW-2 F22, and LH F10, were used for genotyping assays. All strains were identified by 16 S rDNA gene sequencing [32]. The following genotyping methods were performed: MLST [33], P146-based genotyping [34], and MLVA [35]. The same conditions for PCR analysis were used: initial denaturation at 94 °C for 5 min, followed by 40 cycles of denaturation at 94 °C for 1 min, annealing at 48.6–60 °C for 1 min, and extension at 72 °C for 45 s. The final extension step was conducted at 72 °C for 5 min. Table 1 lists primers, annealing temperatures and reaction system composition for three MLST housekeeping genes (*adk*, *rpoB*, and *tpiA*), P146 gene, and 13 MLVA loci. The amplicons obtained were evaluated by 1% agarose gel electrophoresis in TBE buffer (90 mM Tris, 90 mM borate, and 2.5 mM EDTA [pH = 8]). All positive PCR amplification products were sent for sequencing without prior purification (GenScript, Nanjing, China). To ensure reproducibility, analyses were performed three times. The corresponding sequences of strains 232 (NC_006360.1), 7448 (NC_007332.1), and 7422 (NC_021831.1) were obtained from the whole genome data in NCBI.

**Table 1:** **
Primers and optimized procedures for MLST, P146 gene and MLVA analyses of *M. hyopneumoniae* isolates**

Methods	Target genes	Sequence (5′→3′)	10×PCR buffer (Mg2 + free, µL)	MgCl_2_ (25mM, µL)	dNTP (2.5mM, µL)	Primer (10µM, µL)	Taq DNA polymerase (5U/µL, µL)	Annealing temperature (℃)	References
MLST	*adk*	GGAGCTCCTGGCTCAGGTAAAG	2	2	2	0.8	0.5	60	[33]
		GTTTCTTCAAGGGTTTGCTCG							
	*rpoB*	AAACGGATAGTTAGTGTTGGCG	2	2	2	0.8	0.5	60	
		TGTTCGGCATCAAGGACAAG							
	*tpiA*	GAAATTGAAAAATGAATAAAACCGTAAG	2	2	2	0.8	0.5	60	
		GATGCTTTTCTGGGATACTAACTCG							
P146 sequencing	P146	TCCAAGACGAAGATCTTGACTATC	2	2	2	0.8	0.5	56	[34]
		TTAGAACTTGCAAGATAAAGCTTG							
MLVA	P97 R1	GAAGCTATCAAAAAAGGGGAAACTA	2	3	3	0.8	0.8	59.7	[11]
		GGTTTATTTGTAAGTGAAAAGCCAG							
	P97 R2	AGCGAGTATGAAGAACAAGAA	2	3	3	0.8	0.8	50.5	
		GGTTTATTTGTAAGTGAAAAGCCAG							
	H2R2	TCAATAACAAAGCACCTTTC	2.5	2	2	1.1	0.8	50.3	[8]
		GAGCTATTAATCCAGGCATC							
	H3	ATTACCGGAAATAAGAAGTG	2	2.5	2.5	0.8	0.8	55.9	
		AGTTAAAAAAGCGGCTTTTC							
	H5R1/R2	AAGTAAAAAAGACCTCGAAG	2	2.5	2	1.4	0.8	50.3	
		CCAAAGAATTAATCCAAGTC							
MLVA	H6R3	CAGATCAAATGGCTGTAACA	3	1.5	2.5	1.1	0.8	59.7	Designed in this study
		CCTTCACGGATTGCTTCA							
	CH	GGCAAAAAAATGTGAATGTC	2	2	2	1.1	1.1	52.4	[8]
		GGCTTTTTGGTATATTCAGTTC							
	P95	ATTTATCCTTTACTTAGCGG	2.5	2	2	1.4	0.8	50.3	
		AAGACAAGTGGATATTTTGC							
	P146R1	AGTCACAAAAACCTCAAAGTG	2	2	2	1.1	0.8	52.3	
		AGGAGAGCTTTGAATTTGAG							
	P146R2	AAGACCAAAAAGTAGGACATAC	3	2	2	1.1	1.1	48.6	
		AACTCAAGCATCTAAAAGTG							
	P216	GCAAAGCCAAAATGTAAATG	2	2	2	0.8	0.8	52.4	
		CCAGTTCTTCTTCTTTTCTAAC							
	P146R3	AAAACCCAAAGTAGTGATTC	2	2	2	1.1	0.8	50.3	
		TGTATCGGTTTCAGAAGAAG							

#### Clustering and discriminatory power analyses of the genotyping methods

MLST allows the analysis of clonal origin of strains and the phylogenetic relationship between them by indexing nucleotide variation. Sequences of the three housekeeping genes *adk*, *rpoB*, and *tpiA* were submitted to the MLST database. Each unique gene sequence was assigned an allele number, and the combination of three allele numbers for each strain defined the sequence type (ST) [33]. Likewise, each of the 10 strains corresponded to a P146-based genotype in the MLST database according to the P146 gene sequencing results [34]. VNTR for each strain were determined from the number of repeats in the amplicons. Data generated by the three genotyping methods were analyzed with BioNumerics® 7.5 (Applied Maths, Belgium). Categorical coefficients were used for defining similarity levels and unweighted pair group method with arithmetic mean (UPGMA) was used as the clustering algorithm [36]. The dendrogram and minimum spanning tree thus obtained were used to verify genetic relationships among strains. Using a set of pairwise distances that describe the degree of dissimilarity among strains, a minimum spanning tree represents a set of edges (connections) that link nodes (strains) by the shortest possible distance [37]. The Simpson index of diversity was used to determine and compare the discriminatory power of the three genotyping methods according to the formula described by Hunter and Gaston [38]:

$$D=1-\frac{1}{(N(N-1))}\sum\limits_{j=1}^{s}{{{n}_{j}}}({{n}_{j}}-1)$$

D = Simpson index of diversity, N = total number of samples, s = total number of types described, and nj = number of strains belonging to the j type.

### Biofilm formation by *M. hyopneumoniae* strains

The ability of *M. hyopneumoniae* strains to form biofilms was assessed using a previously reported method by Petrelli et al. [39], with slight modifications. Biofilm cultivation was initiated when the activity of *M. hyopneumoniae* strains reached 10^8^ CCU/mL. Bacterial culture (200 µL) was placed into a 12-well glass bottom plate (Costar, Corning Inc., USA) filled with 1800 µL of fresh KM2 medium, followed by incubation at 37 ℃ for 1 day under static conditions. To extend the growth time, the medium was changed daily. Supernatant (1800 µL) was discarded and the same volume of fresh KM2 medium was added. Biofilm formation was monitored after incubation for 1, 2, 3, 4, and 5 days, as described below. The plates were gently washed thrice with 2.5 mL sterile PBS to remove planktonic bacteria. The biofilms were fixed with 2.5 mL methanol for 15 min at room temperature and stained with 2.5 mL 1% crystal violet for 5 min. After washing thrice with 2.5 mL sterile PBS and observing morphological changes under a microscope (Zeiss Axio Vert, SY-04, Germany), the samples were dissolved in 2.5 mL 95% ethanol for 10 min until crystal violet on the glass surface had completely dissolved. The absorbance of 125 µL eluent was measured at 570 nm (OD_570_) (BioTek ELx800; BioTek Instruments Inc., Winooski, VT, USA). The experiment was conducted in triplicate, and uninoculated medium was used as the NC.

### Correlation analysis between strain virulence, genotypes, and biofilm formation ability

Binary logistic regression analysis was used to evaluate virulence-associated factors, including genotypes determined by MLST, P146-based genotyping, and 13 MLVA loci using the IBM SPSS Statistics software (v 23.0). The dependent variable is usually a dummy variable with values. With regards to virulence, 0 represented low and 1 represented high virulence. Further, Pearson correlation analysis was also used to evaluate the association between biofilm formation ability and virulence. Microtiter plate biofilm assay (OD_570_) was performed to determine biofilm formation ability. Virulence was assessed by determining the ratio of dead to live mucosal epithelial cells (R/G ratio). The correlation between OD_570_ and R/G ratio was calculated using the methods mentioned in the section “Assessing the ability of the strains to infect the tracheal mucosa”.

## Results

### Assessment of virulence of *M. hyopneumoniae* strains

A total of 18 *M. hyopneumoniae* antibody-negative Bama miniature piglets were selected for challenge (Additional file 1). After challenge with 5 different *M. hyopneumoniae* strains via the tracheal route, various clinical signs including apathy, loss of appetite, coughing and wheezing were observed (Additional file 2).

#### Anatomic observation

**Figure 1:**
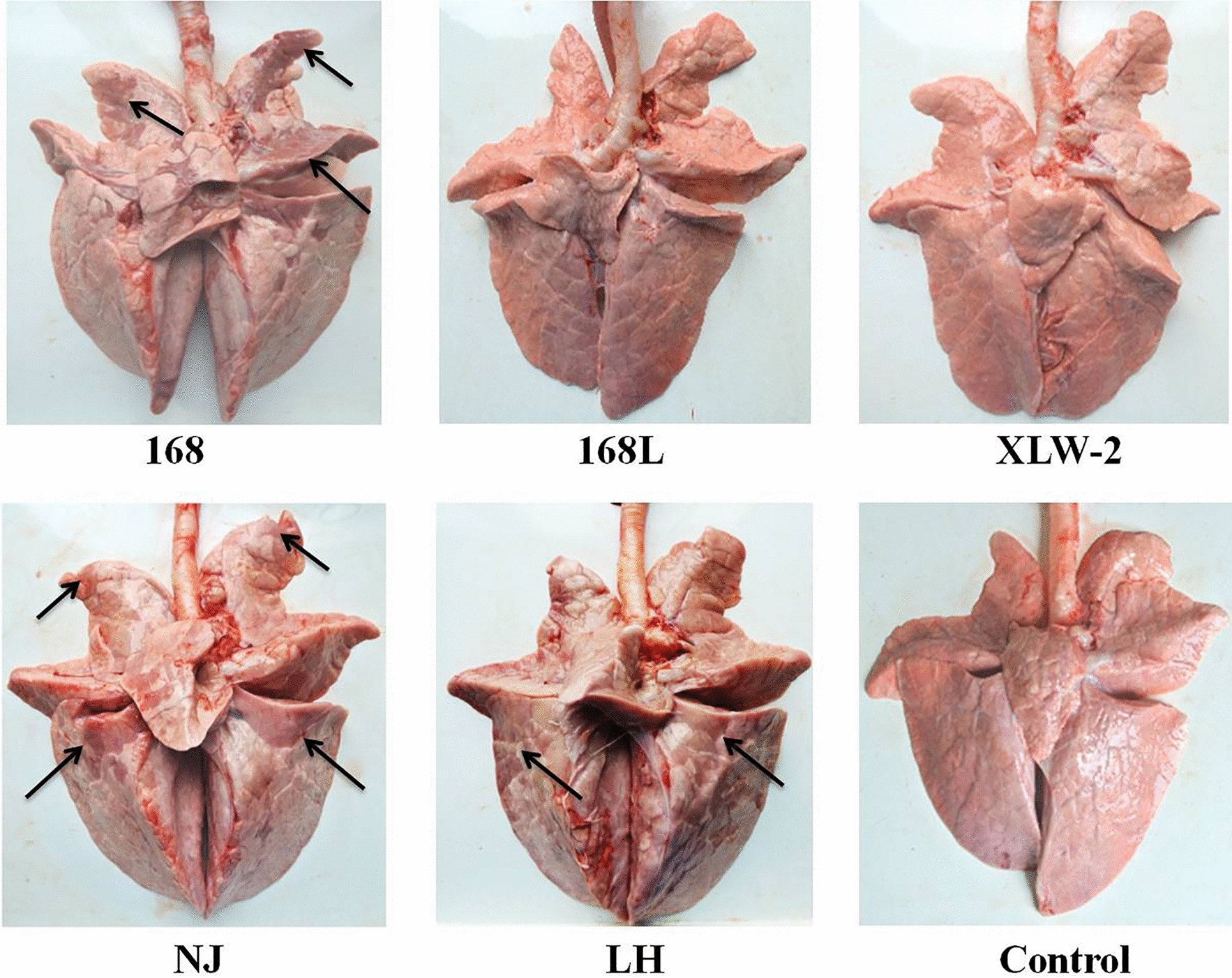
**Anatomical observation of pneumonic lung lesions in** ***M. hyopneumoniae*-infected pigs.** Lungs lesions on infection by strains 168, 168 L, XLW-2, NJ, and LH are shown. Lung lesions with clear boundaries are indicated by black arrows. Control: lungs without any infection.

Pigs were euthanized 28 days post-challenge. After necropsy, anatomical observation of the lungs of pigs infected with strains 168, NJ, and LH demonstrated typical MPS lesions with dark red–purple areas of pulmonary consolidation. Lung lesions show clear boundaries. Gross lung lesions, reflecting the levels of pathological changes in various parts of the lung (proportion of consolidation), were evaluated using the 28-point evaluation method (Figure 1; Table 2). The mean lung lesion scores were 10.5 for the 168-challenge group, 10.17 for the NJ-challenge group, 12.33 for the LH-challenge group, 0.33 for the 168 L-challenge group, and 0 for the XLW-2-challenge and NC groups. Statistical analyses demonstrate that strains 168, NJ, and LH show significantly stronger pathogenicity than strains 168 L and XLW-2. Further, strains 168 L and XLW-2 show low or no virulence.

**Table 2:** **Pneumonic lung lesions in *M. hyopneumoniae* infected pigs evaluated using the 28-point evaluation method**

Group	No.	Left apical lobe	Left cardiac lobe	Left diaphragmatic lobe	Right apical lobe	Right cardiac lobe	Right diaphragmatic lobe	Intermediate lobe	Total score	Average lung lesion score ± SD	Difference compared with the control group
168	1-1	0	4	0	2	2	1	2	11	10.5 ± 2.78	
	1-2	0	0.5	0.5	1	4	0.5	1	7.5		**
	1-3	4	4	1	1	1	1	1	13		
168 L	2-1	0	0	0	0	0	0	0	0		
	2-2	0	0	0	0	0	0	0	0	0.33 ± 0.58	–
	2-3	0.5	0	0	0.5	0	0	0	1		
XLW-2	3-1	0	0	0	0	0	0	0	0		
	3-2	0	0	0	0	0	0	0	0	0 ± 0	–
	3-3	0	0	0	0	0	0	0	0		
NJ	4-1	2	2	0.5	2	3	1	1.5	12		
	4 -2	0	4	1	3	0.5	1	3	12.5	10.17 ± 3.62	**
	4-3	1	1	1	0	1	1	1	6		
LH	5-1	1	1	1	4	2	0	1	10		
	5 -2	4	4	0	3	4	1	0	16	12.33 ± 3.21	**
	5-3	2	2	1	2	2	1	1	11		
Control	6 -1	0	0	0	0	0	0	0	0		
	6-2	0	0	0	0	0	0	0	0	0 ± 0	-
	6 -3	0	0	0	0	0	0	0	0		

“**” indicates significant difference.

“–” indicates no significant difference.

#### Pathohistological observation

**Figure 2:**
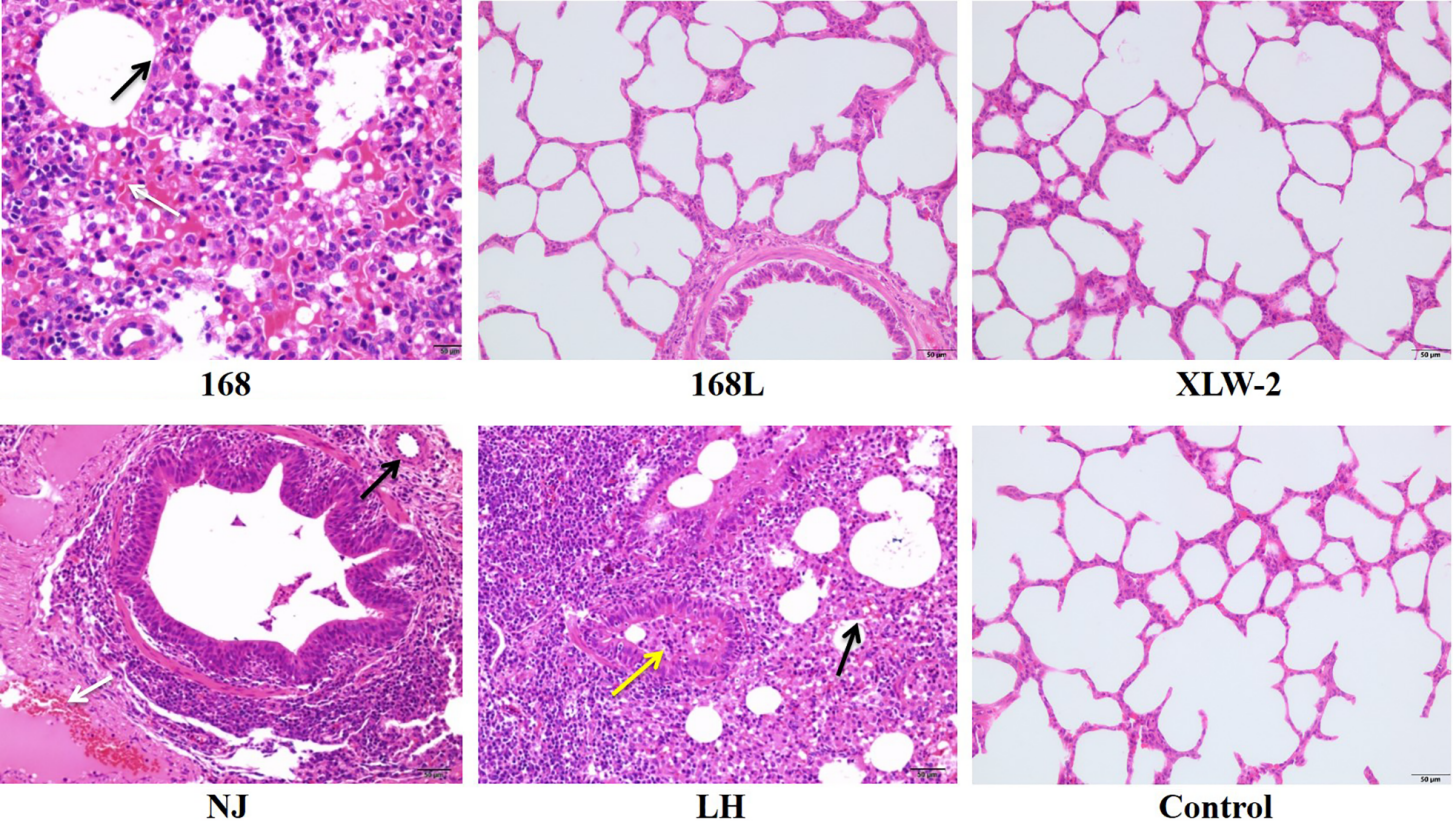
**HE staining showing pathological changes in** ***M. hyopneumoniae*-infected pig lung tissues.** 168, 168 L, XLW2, NJ, and LH: pathological changes on infection with the corresponding strain. Control: negative control. The swelling and hyperplasia of alveolar epithelial cells are indicated by black arrows. Smaller or occluded alveolar lumen and narrowed bronchial lumen infiltrated with inflammatory and deciduous epithelial cells are indicated by yellow arrows. Congestion or hemorrhage around alveoli and bronchi is indicated by white arrows.

To further investigate the pathological changes induced by *M. hyopneumoniae* infection, lung tissues of challenged as well as unchallenged animals were analyzed by hematoxylin–eosin (HE) staining. As shown in Figure 2, in comparison with the NC groups, the NJ-, 168-, and LH-challenge groups show lung lesions of unclear tissue structure and thickened alveolar walls, indicating the occurrence of pneumonia. The swelling and hyperplasia of alveolar epithelial cells resulted in smaller or occluded alveolar lumen and narrowed bronchial lumen. Further, in the LH-challenge group, infiltration with inflammatory and deciduous epithelial cells was frequently observed. In the 168- and NJ-challenge groups, some evidence of congestion or hemorrhage around alveoli and bronchi was observed. The NJ-, 168- and LH-challenge group show more serious alveolar epithelial cell hyperplasia, resulting in nearly no visible alveolar structures. In contrast, the 168 L- and XLW-2-challenge groups showed no obvious microscopic lesions. Quantitative analyses of *M. hyopneumoniae* in lung DNA samples show that the bacterial load of strains 168, NJ, and LH was more than that of strains 168 L and XLW-2 (Additional file 2). To summarize, animal challenge experiments indicate that 168, NJ, and LH show high virulence, while 168 L and XLW-2 show low virulence (Table 2).

#### Assessment of *M. hyopneumoniae* virulence using in vitro tracheal mucosa infection tests

**Figure 3:**
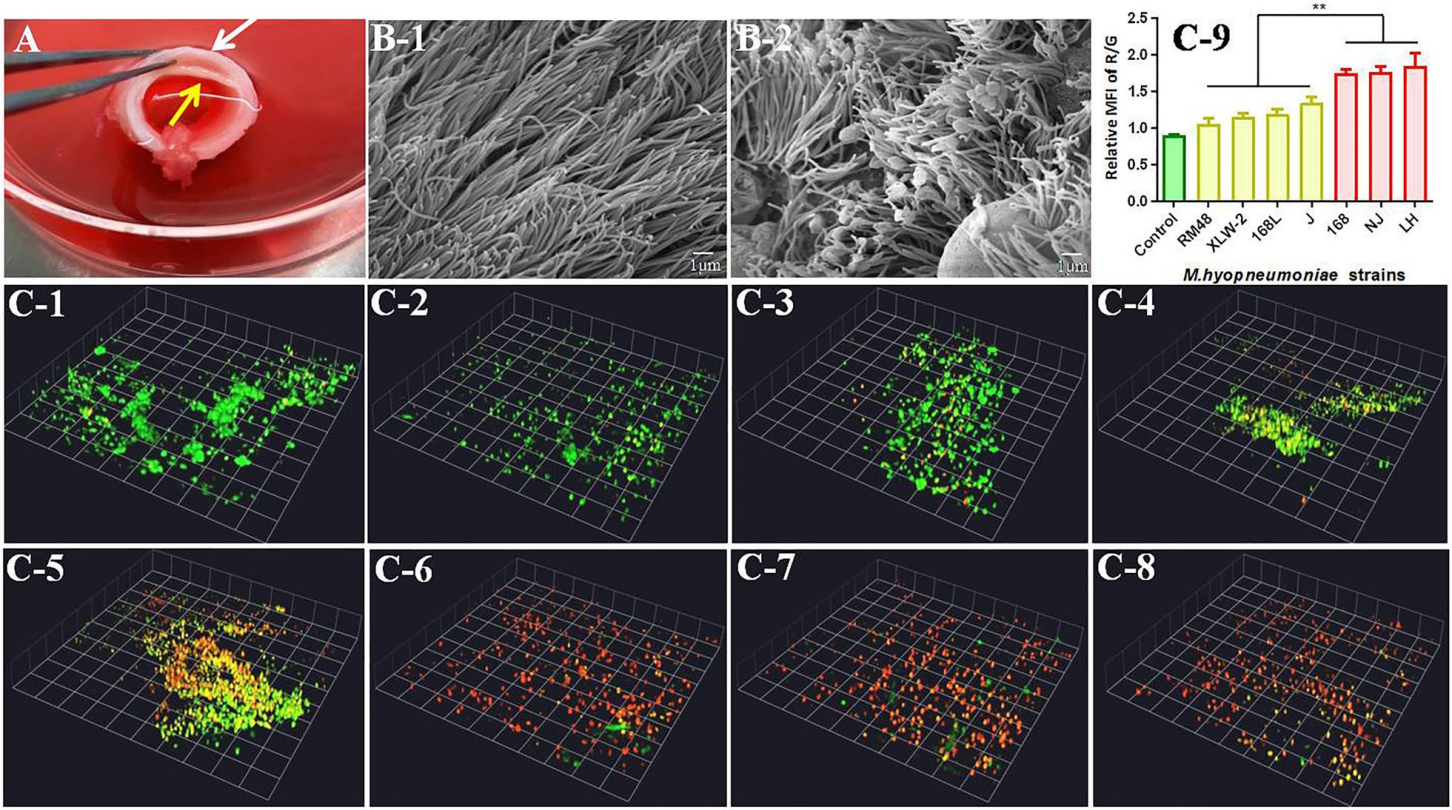
**Damage caused by** ***M. hyopneumoniae*** **infection to porcine tracheal epithelial cells.** **A** Tracheal mucosa preparation. The cartilage (white arrow) was excised to reserve the inner tracheal mucosa (yellow arrow) for in vitro tracheal mucosa infection tests. **B** Scanning electron microscopy showing damage to porcine tracheal epithelial cilia. **B** Panel 1: negative control, with no damage. **B** Panel 2: Porcine tracheal epithelial cells after infection with strain 168 for 24 h. **C** Laser scanning confocal microscopy showing viability of tracheal epithelial cells on infection with different *M. hyopneumoniae* strains for 24 h. Dead cells emitted red fluorescence, while live cells emitted green fluorescence. The thickness of the vertical structure of cells was analyzed by the x-y-z axis scanning with confocal microscopy. Three-dimensional diagrams of *M. hyopneumoniae*-infected and control cells are shown. **C** panel 1: negative control, **C** panel 2: RM48, **C** panel 3: XLW-2, **C**, panel 4: 168 L, **C**, panel 5: J, C, panel 6: 168, **C** panel 7: NJ, and **C** panel 8: LH. **C** panel 9: comparison of cell viability. Values represent the ratio of mean fluorescence intensity of red fluorescence relative to green fluorescence.

To further evaluate the virulence of *M. hyopneumoniae* strains RM48 and J and to parallelly compare the virulence of the aforementioned seven strains, we used the tracheal mucosa tissue (Figure 3A), which is the first colonization site of *M. hyopneumoniae*. After incubation with various *M. hyopneumoniae* strains for 24 h, scanning electron microscopy shows that *M. hyopneumoniae* cells mostly colonized the apical tips of cilia and induced disturbance and loss of cilia, suggesting that *M. hyopneumoniae* caused cytotoxic damage to tracheal mucosal epithelium (Figure 3B, panel 2). The uninfected tracheal mucosa demonstrates intact cell surface and neatly organized cilia (Figure 3B, panel 1).

To further quantify the detrimental effects of *M. hyopneumoniae* strains, we stained *M. hyopneumoniae*-infected tracheal mucosa, followed by observation under a laser scanning confocal microscope. Cells with intact membranes appeared fluorescent green, whereas those with damaged membranes appeared fluorescent red (Figure 3C, panels 1–8). As evident from Figure 3C, panel 9, the R/G ratio of cells incubated with strains LH, NJ, and 168 was significantly higher than that of other strains, indicative of severe cell damage. Strains J, 168 L, XLW-2, and RM48 caused lesser cell death, implying that the decrease in cell viability caused by strains LH, NJ, and 168 was higher than that by strains J, 168 L, XLW-2, and RM48. This indicated that the virulence of strains LH, NJ, and 168 was higher than that of strains J, 168 L, XLW-2, and RM48. Table 3 shows comprehensive analyses of the virulence of all 10 *M. hyopneumoniae* strains. Animal challenge experiments and in vitro tracheal mucosa infection test results show good consistency. The association between virulence as determined by animal challenge experiments and results of in vitro tracheal mucosa infection test of strains 168, 168 L, LH, NJ, and XLW-2 was further evaluated with the Pearson correlation analysis. The r value between lung lesion score and R/G ratio of these strains was 0.971 (*p* < 0.01), indicative of a strong correlation (Additional file 3).

**Table 3:** **
Comprehensive analysis of virulence of *M. hyopneumoniae* strains following challenge experiments**

Source	Strains	Geographical origins	Virulence	Challenge tests^*^	References
International strains	J	Brazil	Low	×	[21]
	232	USA	High	×	[23]
	7448	Brazil	High	×	[24]
	7422	Brazil	High	×	[24]
Vaccine strains	RM48	China	Low	×	–
	168 L	China	Low	√	This study
Field isolates	168	China	High	√	This study
	NJ	China	High	√	This study
	XLW-2	China	Low	√	This study
	LH	China	High	√	This study

√: Challenge tests shown in this study.

×: Challenge tests not performed in this study.

### MLST, P146-based genotyping, and MLVA

**Figure 4:**
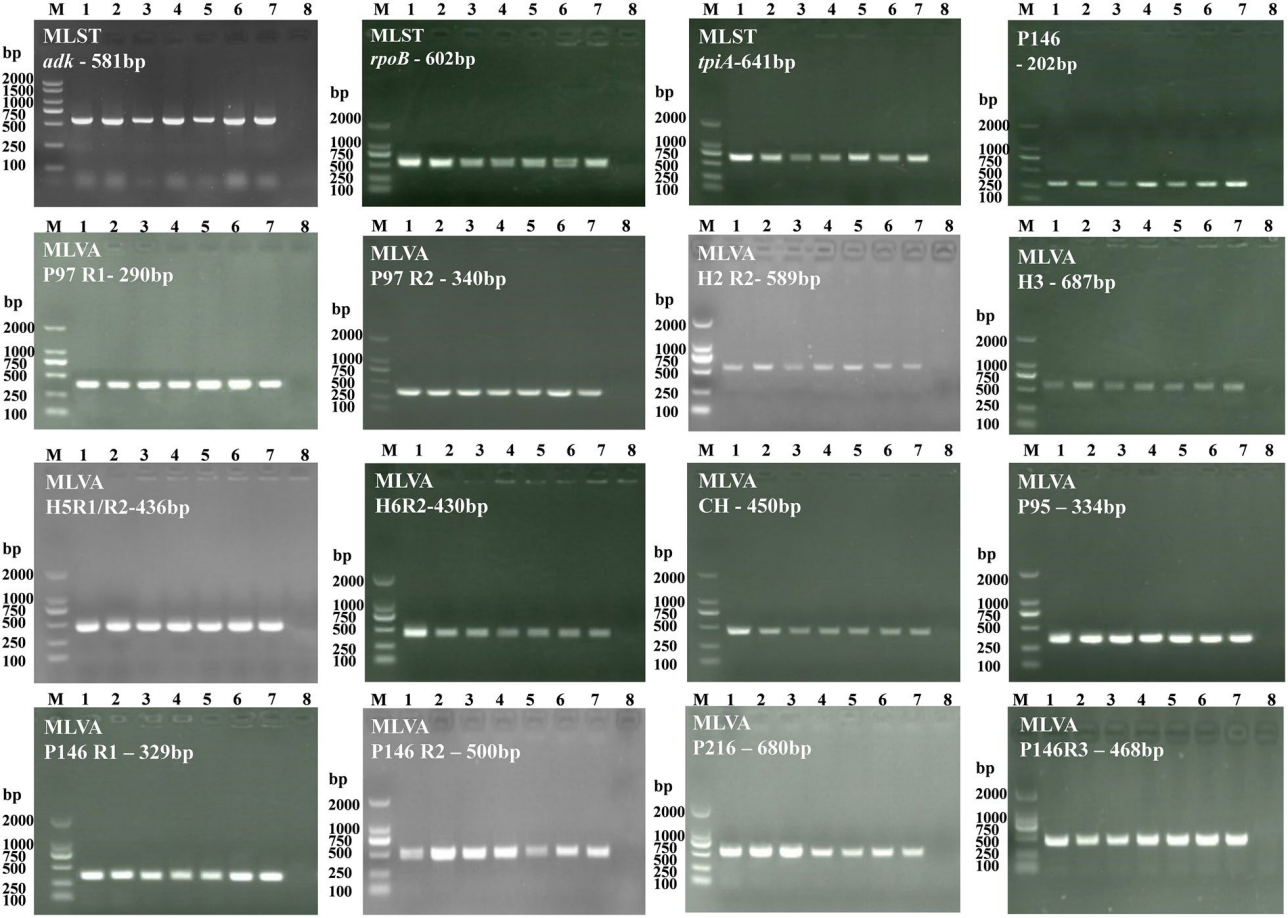
**Agarose gel electrophoresis of amplicons.** The agarose gel electrophoresis of amplicons was obtained on using primers targeting three MLST housekeeping genes (*adk*, *rpoB*, and *tpiA*), P146 gene, and 13 MLVA loci (P97 R1; P97 R2; H2 R2; H3; H5R1; H5R2; H6R3; CH; P95; P146 R1; P146 R2; P146 R3; and P216R1). Lane M: DL 2000 DNA marker; lanes 1–7: strains J, 168, 168 L, NJ, XLW-2, LH, and RM48, respectively; lane 8: negative control.

**Figure 5:**
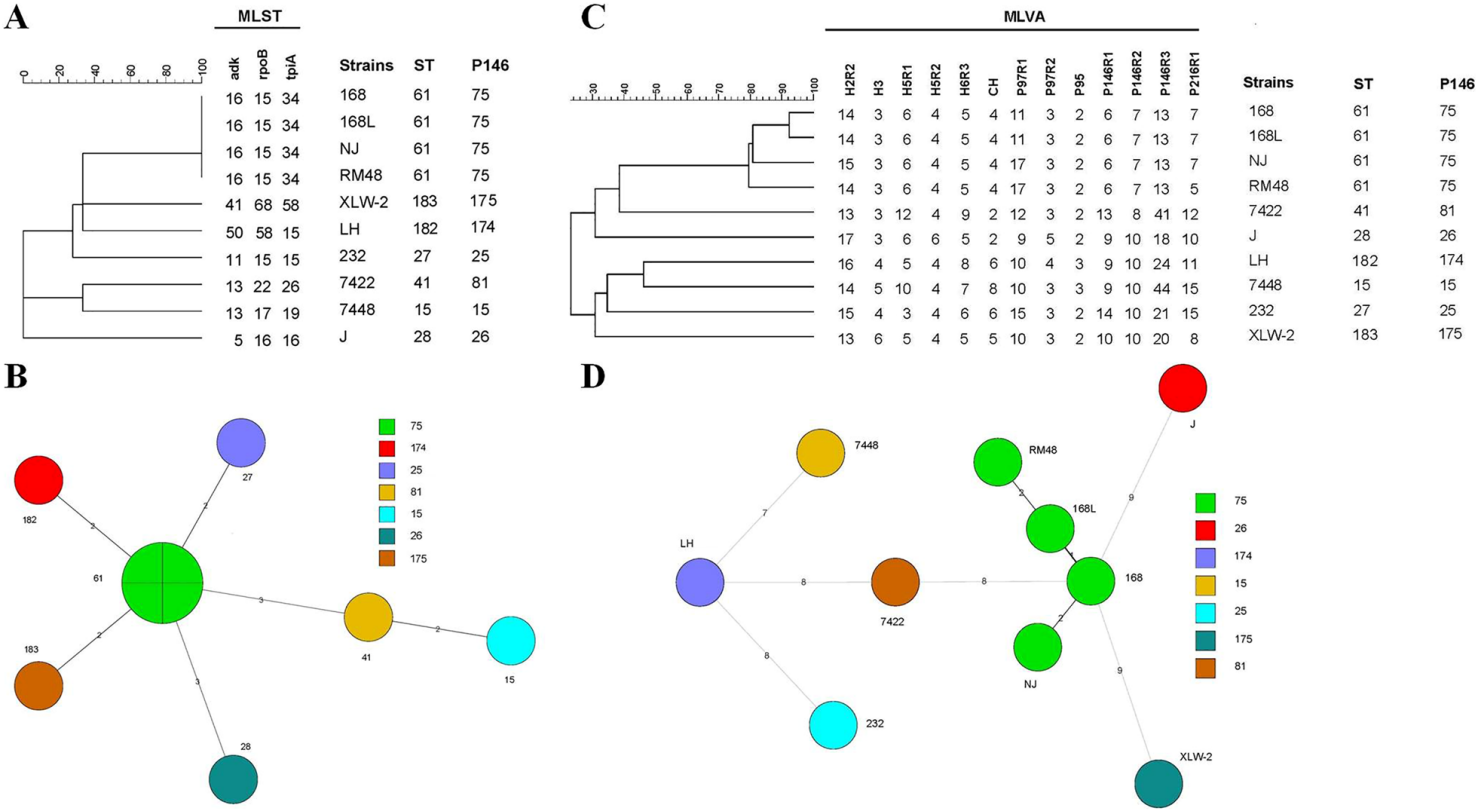
**MLST-, P146-, and MLVA-based dendrograms.** **A** MLST- and P146-based dendrograms. The dendrogram was derived from combining individual distance matrices of *adk*, *rpoB*, and *tpiA* sequences. Strains 168, 168 L, NJ, and RM48 show identical ST (green node in **B** and **D**), and the remaining six strains were clustered into a different ST. P146 ST are appended to the right and demonstrate consistency in allele differentiation. **B** Minimal spanning tree calculated with P146 allelic profiles. Samples in the same color belong to the same P146-based genotype. The numbers around the nodes indicate ST derived by MLST. The allelic profiles and P146-based genotyping classified the 10 strains into seven ST. **C** Dendrogram derived from UPGMA cluster analysis of MLVA profiles obtained using 13 loci. Strains could be delineated into two major clusters, with < 30% similarity. ST and P146-based genotypes are appended to the right and demonstrate consistency in allele differentiation. **D** Minimal spanning tree of all samples with a complete MLVA profile. Genetic distances among strains NJ, RM48, 168 L, and 168 based on MLVA data were closer than among 7422, XLW-2, and J. Strains LH, 7448, and 232 show relatively large genetic distances with other strains. Samples in one color belong to the same P146-based genotype. MLST data were consistent with P146-based genotyping data.

The amplicons obtained on performing PCR with primers for three MLST housekeeping genes (*adk*, *rpoB*, and *tpiA*), P146 gene, and 13 MLVA loci for strains J, 168, 168 L, NJ, XLW-2, LH, RM48 were subjected to agarose gel electrophoresis. As shown in Figure 4, specific bands representing amplicons of different sizes were observed. The primers show good specificity. The amplicons were subsequently subjected to sequencing (Additional file 4), and MLST analysis was performed using sequencing data. As shown in Figures 5A and C, each unique gene sequence was assigned an allele number, and the combination of three allele numbers for each strain defined the ST. P146-based genotypes were also confirmed according to the MLST database, and corresponding DNA sequences of international reference strains (232, 7448, and 7422) were obtained from NCBI. Finally, the 10 strains were classified as having seven different ST, seven P146-based genotypes, and 10 MLVA types. It is notable that strains 168, 168 L, NJ, and RM48 belonged to the same ST, i.e., ST61, and the same P146-based genotype, i.e., 75.

#### Cluster analysis of MLST, P146-based genotyping, and MLVA data

Sequences obtained from NCBI and sequencing data of three MLST housekeeping genes (*adk*, *rpoB*, and *tpiA*) for strains J, 168, 168 L, NJ, XLW-2, LH, RM48, 232, 7448, and 7422 were analyzed using BioNumerics®. We performed UPGMA cluster analysis, dendrogram construction, and minimum spanning tree analysis. As shown in Figure 5A, based on the analysis involving the MLST database, strains RM48, 168 L, 168, and NJ (isolated in China) were clustered in one branch and show a closer genetic relationship with strains XLW-2 and LH (isolated in China) than with strains 232, 7422, 7448, and J (isolated in the Americas), indicative of significant geographic differences (Table 3). This result was further validated by P146-based genotyping data. Minimum spanning trees are frequently used in molecular epidemiology research to estimate relationships between individual isolates. The different node colors shown in Figure 5B represent seven different ST/P146-based genotypes. Four strains (168, 168 L, NJ, and RM48) show identical ST/P146-based genotypes (green node), while the remaining six were clustered into different ST/P146-based genotypes. Strains RM48, 168 L, 168, and NJ were more similar to strains XLW-2, 232 and LH than to strains J and 7422, while the most dissimilarity was observed with strain 7448.

VNTR varied depending on loci and strains. As shown in Additional file 4 and Figure 5C, repeat number variation was predominantly observed for the genetic loci P97 R1, P146 R3, and P216 R1. Based on the MLVA-derived dendrogram, there were two major clusters with < 30% relatedness (Figure 5C). Strains 168 and 168 L appeared in one branch and revealed a close genetic relationship, with variations only observed in the VNTR of P146 R1. Although the VNTR of P146R1 in strains 168 and 168 L were the same, the specific repeat region in strains 168 L and 168 was “QPQ” and “PQ”, respectively (Additional file 4). This can be explained by the fact that strain 168 L is a highly passaged, attenuated strain derived from the pathogenic strain 168. In addition, strains NJ, RM48, 168 L, and 168 were clustered together and show approximately 80% homology. The second cluster comprised strains LH, 7448, 232, and XLW-2, demonstrating 30–50% relatedness. The genetic relationships among the various strains were further clearly illustrated based on MLVA profiles and the minimum spanning tree (Figure 5D). Strains 168 L, 168, NJ, and RM48, identified as having the same P146-based genotype as well as the same ST, were significantly different from the other six strains.

#### Discriminatory power analysis of different genotyping methods

The Simpson index of diversity was used for the discriminatory power analysis of the three genotyping methods. The 10 strains were separated into seven clusters by MLST and P146-based genotyping. The Simpson index of diversity for MLST and P146-based genotyping was 0.867. Ten MLVA types were identified in total. All strains were considered typeable by MLVA. The Simpson index of diversity for MLVA was calculated to be as high as 0.978, higher than that for MLST and P146-based genotyping, indicating a higher discriminatory power.

### Analyzing the correlation between genotyping and virulence

Based on MLST and P146-based genotyping, strains RM48, 168 L, 168, and NJ were assigned to the same ST. However, strains RM48 and 168 L show low virulence, while 168 and NJ show high virulence. Strains XLW-2 (low virulence) and LH (high virulence) were closely related to the four aforementioned strains. Binary logistic regression analysis was used to elucidate the correlation between different genotyping analyses and *M. hyopneumoniae* virulence. Neither MLST nor P146-based genotyping data revealed any significant differences in the virulence of the 10 strains (*p* > 0.05) (Table 4). Based on MLVA, the attenuated strain 168 L and the virulent strain 168 shows a close genetic relationship. The attenuated strain RM48 and the virulent strain NJ were more closely related to strains 168 and 168 L. Strains LH and 232 are both highly virulent, but shared a distant relationship with highly virulent strains 168 and NJ. It is notable that based on binary logistic regression analysis, differences in the virulence of the 10 strains could not be linked to MLST, P146-based genotyping, or MLVA profiles (*p* > 0.05) (Table 4).

**Table 4:** **Binary logistic regression analysis of the virulence and MLST, P146, MLVA for 10 strains**

Variable		Beta	*P* value*	OR	OR 95% CI^#^
MLST	ST	−0.023	0.583	0.977	0.900-1.061
P146	P146-based genotypes	−0.006	0.812	0.994	0.945–1.045
MLVA	H3	−0.082	0.898	0.921	0.264–3.215
	H5 R1	0.239	0.453	1.270	0.680–2.372
	H5 R2	−0.102	0.886	0.903	0.224–3.639
	H2 R2	−0.274	0.644	0.760	0.238–2.428
	H6 R1	10.490	1.000	1.000	0.000-
	CH	0.475	0.296	1.608	0.660–3.921
	P97 R1	0.150	0.550	1.162	0.711–1.899
	P97 R2	−10.948	1.000	1.000	0.000–
	P95	21.203	0.999	0.999	0.000–
	P146 R1	0.028	0.940	1.029	0.493–2.144
	P146 R2	0.000	1.000	1.000	0.282–3.544
	P146 R3	0.140	0.289	1.150	0.888–1.490
	P216 R1	0.470	0.144	1.600	0.852–3.003

* *P* > 0.05 indicates a non-significant result, while *P* ≤ 0.05 shows a significant result.

# 95% CI: 95% confidence intervals.

### Correlation analysis between virulence and biofilm formation ability

**Figure 6:**
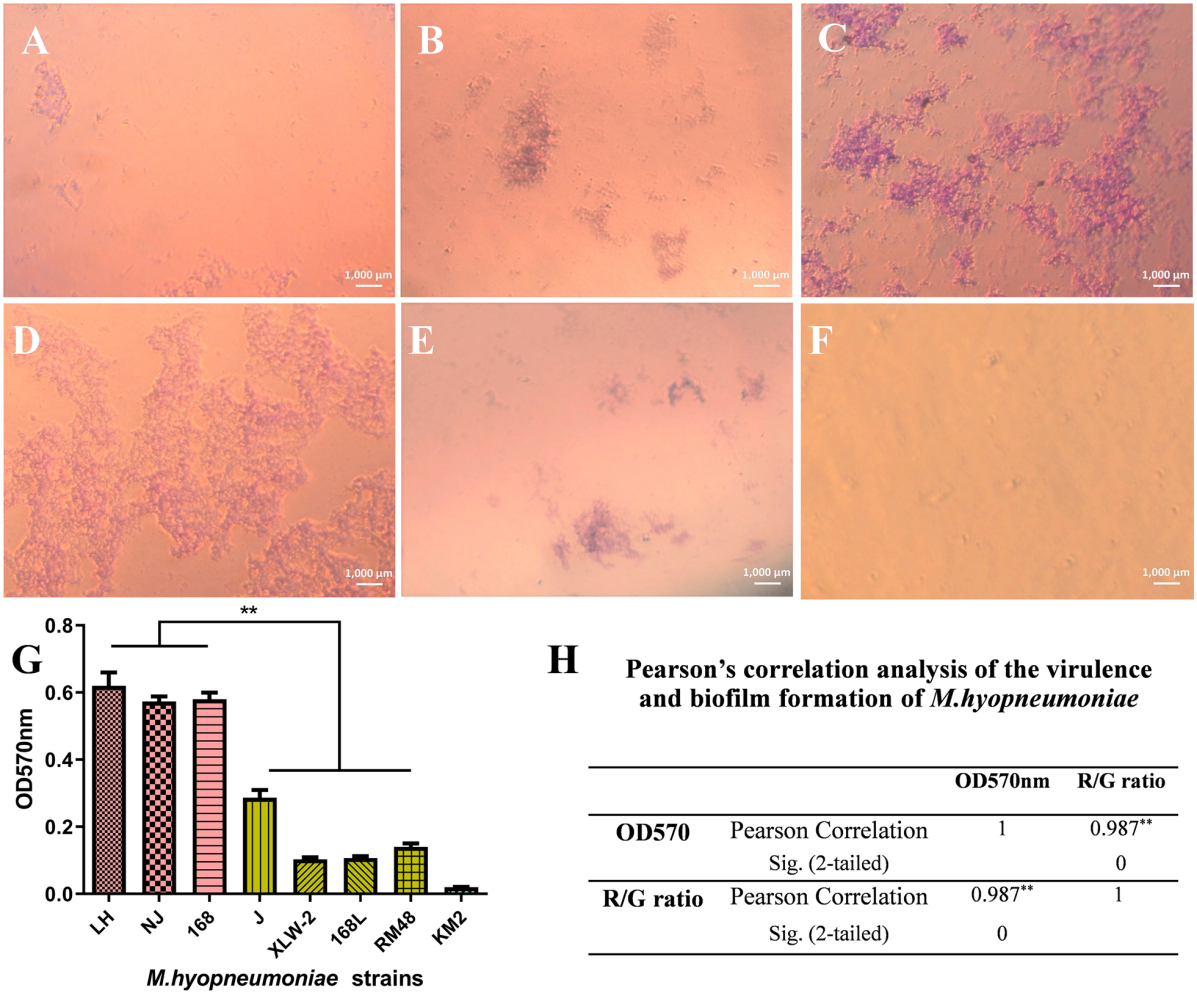
**Biofilm formation ability of** ***M. hyopneumoniae*** **strains.** **A**–**F** Biofilms formed by strain NJ, which were stained with crystal violet and observed under a microscope after **A** 1 day, **B** 2 days, **C** 3 days, **D** 4 days, and **E** 5 days of incubation. **F** negative control. **G**, **H** Comparison of biofilms formed by seven *M. hyopneumoniae* strains. Biofilm formation ability of strains was determined and identified by crystal violet staining after 4 days of incubation. Bars represent mean ± SD for three independent replicate experiments. KM2 represents control (uninoculated broth).

To minimize our dependency on animal challenge experiments, which are expensive, complex, and time consuming, we further assessed the virulence of seven *M. hyopneumoniae* strains by determining the correlation between their virulence and ability to form biofilms. To understand the pattern of biofilm formation, we randomly selected the highly virulent *M. hyopneumoniae* strain NJ. After crystal violet staining, bacterial adsorption and single colony formation were observed from the first day of seeding. On the second day of culture, the number of bacterial colonies gradually increased. After culturing for 3 to 4 days, the colonies gathered together to form mucoid colonies, and biofilm thickness was observed to increase. On the fifth day of culture, part of the biofilm began to dissipate. Thus, the optimal biofilm culture time was determined to be around 3 to 4 days (Figures 6A–F).

Mature biofilms of more strains were then assessed after 4 days of culture. All the seven strains (168, 168 L, NJ, XLW-2, RM48, J, and LH) could form biofilms, with average OD_570_ being 0.107 ± 0.005–0.633 ± 0.004 (Figure 6G). The highly virulent strains 168, NJ, and LH possessed a strong ability to form biofilms, while the attenuated strains J, 168 L, XLW-2, and RM48 show a relatively weak ability to form biofilms. Biofilm formation ability, indicated by OD_570_, and virulence, and by the R/G ratio of *M. hyopneumoniae* strains were analyzed using the single sample K-S test. The two variables were normally distributed (*p* > 0.05) and could be analyzed with Pearson correlation analysis (Additional file 5). The r value between OD570 and R/G ratio was 0.987 (*p* < 0.001), which is > 0.8 and thus indicated an extremely strong correlation (Figure 6H). Thus, virulence characteristics of the seven tested *M. hyopneumoniae* strains were found to be highly correlated with their biofilm formation ability.

## Discussion

*Mycoplasma hyopneumoniae* strains show wide differences in their pathogenic effects in pigs. However, little remains known regarding factors responsible for differences in virulence. Herein we aimed to identify factors associated with variations in the virulence of *M. hyopneumoniae* strains. First, the virulence characteristics of a defined set of *M. hyopneumoniae* strains were identified by performing animal challenge experiments, and the results were complemented and re-evaluated with in vitro tracheal mucosa infection tests.

Animal challenge experiments confirmed that strains 168, NJ, and LH were highly virulent, while 168 L and XLW-2 show low virulence. However, individual diversity was observed among the animals. This has also been highlighted by other studies, implying that many factors affect the results of animal challenge experiments [40]. In addition, animal challenge experiments are associated with animal welfare concerns, plus they are time consuming, complex to perform, and expensive. To comprehensively evaluate the virulence of some strains without involving animals and to parallelly compare the virulence characteristics of more *M. hyopneumoniae* strains, we constructed a swine tracheal mucosa infection model. *M. hyopneumoniae*-infected trachea had significantly increased levels of tracheal ciliary injury. Cell viability assay was performed to quantitate cellular injury; the extent of cell death caused by different strains was in the following order from high to low: LH, NJ, 168, J, 168 L, XLW-2, and RM48. The level of damage caused by *M. hyopneumoniae* to ex vivo tracheal mucosa was similar to that observed in animal challenge experiments, indicating the correlation between this approach and *M. hyopneumoniae* virulence. However, more studies, including experimental infections in pigs, need to be conducted to validate our preliminary findings.

To confirm the virulence of the strains used in this study, we investigated the association among virulence, genotypes, and biofilm formation ability. *M. hyopneumoniae* strains demonstrate high gene polymorphism [4, 37]. *M. hyopneumoniae* strains were mainly differentiated by three genotypic methods: MLST, P146-based genotyping, and MLVA. Mayor et al. assayed 54 *M. hyopneumoniae* samples using P146-based genotyping and MLST, and found that MLST showed stronger discriminatory power [33]. Kuhnert et al. investigated 34 lung samples and reported that P146-based genotyping displayed better classification ability [41]. However, in this study, MLST and P146-based genotyping allowed for identical clustering of isolates. Thus, using a combination of these typing methods may not increase the discriminatory ability; their effectiveness may be specific to some groups of strains. Moreover, strains RM48, 168 L, 168, and NJ had the same genotype (ST61), but they show differences in their virulence: 168 and NJ show high virulence, while RM48 and 168 L show low virulence. Furthermore, MLST and P146-based genotyping revealed a close relationship between the highly virulent strains NJ and LH and attenuated strains XLW-2 and RM48. Thus, in this study, we observed lack of correlation between MLST and P146-based genotyping and virulence data. We, however, believe that this lack of correlation is somewhat attributable to the methods used in this study, rather than to the absence of actual differences. For instance, 168 and 168 L show similar genotypes. However, the attenuated virulence of 168 L could be because 168 L is the high-passage strain of 168. These strains show differences in virulence due to differences in genes, but detecting such differences with the methods used in this study is challenging because the three genotyping methods are limited to partial identification of specific genes.

To differentiate strains, for MLVA typing, we chose 13 loci of P97, P146, and P216 considering that they are the key adhesion factors of *M. hyopneumoniae*. Based on our analyses, we suggest that P97 R1, P146 R1, P146 R3, and P216 R1 genes can be preferentially used to characterize clinical samples as they show higher discriminatory ability than other genes. However, variations were observed in the number of repeats between virulent strains, such as 168 and NJ, as well as between strains showing high and low virulence. The P97 R1 repeat region in the vaccine strains 168 L and RM48 was observed to contain 11 and 17 repeats, respectively; the same region in the virulent strains 168 and NJ also contained 11 and 17 repeats, respectively, while that in the virulent strains 232 and LH contained 15 and 12 repeats, respectively. Our results indicate that *M. hyopneumoniae* virulence was not correlated with the number of repeats in the P97 R1 region. Furthermore, it was difficult to correlate virulence with amino acid repeat sequences in P216 and P146 genes. Logistic regression analysis indicates that there was no significant correlation between the virulence of strains examined in this study and MLVA typing.

Strains 232 [42], J [43], 7448 [24], and 7422 [25] were isolated from infected swine in the USA, UK, and Brazil. Based on genotyping data, it appears that the origin of strains isolated from China is distinct from that of those isolated from the USA, UK and Brazil. MLVA typing can help elucidate the phylogenetic relationship among strains, but not virulence, and it is hence useful for epidemiological investigations and tracking of the origin of an outbreak. To date, no association has been found between genotypes and virulence characteristics of the 10 strains included in this study. This could be addressed by assessing a higher number of loci or more virulence-associated genes [3]. Besides, studying a larger number of strains with diverse geographical origins or those from wild vs. domestic pigs may provide better insights into the correlation between genotypes and virulence characteristics. We believe that to elucidate the correlation between genotypes and virulence, techniques such as whole genome sequencing can prove to be highly useful and can also generate a lot more information than P146-based genotyping, MLST, or MLVA.

Biofilms represent one of the main virulence traits associated with the pathogenesis of diverse pathogens [15, 44]. A wide variety of microbes form biofilms, ranging from non-pathogenic to facultative pathogenic species, with distinct virulence potentials [45]. The association between biofilm formation ability and virulence continues to be widely investigated. Different strains reportedly show significant differences in their biofilm formation abilities [46]. However, to date, only a few studies have been conducted on this topic. *M. hyopneumoniae* shows the ability to form biofilms [47]. Herein crystal violet staining revealed that the highly virulent strains 168, NJ, and LH demonstrate strong biofilm formation ability, while the attenuated strains J, XLW-2, 168 L, and RM48 show relatively low biofilm formation ability. Pearson correlation analyses indicate extremely strong correlation between the virulence characteristics and biofilm formation ability of *M. hyopneumoniae*. Thus, we suggest that differences in the virulence of strains can be correlated with their biofilm formation ability. Future studies should assess a higher number of strains to validate the presence of a correlation between biofilm formation ability and virulence. Moreover, we need to enhance our understanding of virulence genes that may be related to biofilm formation ability of various strains.

## Supplementary Information

**Additional file 1. Serological tests of** ***M. hyopneumoniae*** **IgG antibodies for detection of** ***M. hyopneumoniae*** **antibody-negative pigs.**

**Additional file 2. The presence of** ***M. hyopneumoniae*** **in lung tissues (qPCR) and clinical observations of pigs post-infection.**

**Additional file 3. Correction of virulence and results of the in vitro tracheal infection.**

**Additional file 4. Results of DNA sequencing based on MLST, P146 gene sequencing and MLVA.**

**Additional file 5. Correlation of virulence and biofilm.**
