# Supplementary material for: Genotyping and biofilm formation of Mycoplasma hyopneumoniae and their association with virulence
Source: Vet Res. 2022 Nov 17;53:95. doi: 10.1186/s13567-022-01109-x (PMC9673451; doi:10.1186/s13567-022-01109-x)
Supplement: Supplementary file 1 — Additional file 1. Serological tests of M. hyopneumoniae IgG antibodies for detection of M. hyopneumoniae antibody-negative pigs. [file 13567_2022_1109_MOESM1_ESM.docx]

**Additional file 1. Serological tests of *M. hyopneumoniae* IgG antibodies for detection of *M. hyopneumoniae* antibody-negative pigs^a^**

| **Number of samples** | **NC^b^** | **PC^c^** | **S/P** |
| --- | --- | --- | --- |
| 1 | 0.077 | 0.530 | 0.046 |
| 2 |  |  | 0.041 |
| 3 |  |  | 0.023 |
| 4 |  |  | 0.023 |
| 5 |  |  | 0.027 |
| 6 |  |  | 0.030 |
| 7 |  |  | 0.053 |
| 8 |  |  | 0.021 |
| 9 |  |  | 0.103 |
| 10 |  |  | 0.014 |
| 11 |  |  | 0.023 |
| 12 |  |  | 0.023 |
| 13 |  |  | 0.009 |
| 14 |  |  | 0.021 |
| 15 |  |  | 0.000 |
| 16 |  |  | 0.057 |
| 17 |  |  | 0.041 |
| 18 |  |  | 0.037 |

^a^ Validity criteria: the mean optical density (OD_650_) of two positive controls minus the mean OD_650_ of two negative controls ≥ 0.150; the mean OD_650_ of two negative controls ≤ 0.150. The simple calculation method and determination of inspection results was as follows: S/P= (Sample Mean - the mean OD_650_ of two negative controls)/ (the mean OD_650_ of two positive controls minus the mean OD_650_ of two negative controls). A positive result was indicated by an S/P of sample > 0.40; a negative result had an S/P of < 0.30; Doubtful: 0.30≤P/PK≤0.40. The tested serum samples with an OD_650_ < 0.30, judged as negative, could be used as negative animal models.

^b^ NC: the mean OD_650_ of two negative controls.

^c^ PC: the mean OD_650_ of two positive controls.
