# Supplementary material for: Genotyping and biofilm formation of Mycoplasma hyopneumoniae and their association with virulence
Source: Vet Res. 2022 Nov 17;53:95. doi: 10.1186/s13567-022-01109-x (PMC9673451; doi:10.1186/s13567-022-01109-x)
Supplement: Supplementary file 2 — Additional file 2. The presence of M. hyopneumoniae in lung tissues (qPCR) and clinical observations of pigs post-infection. [file 13567_2022_1109_MOESM2_ESM.docx]

**Additional file 2 The presence of *M. hyopneumoniae* in lung tissues (qPCR) and clinical observations of pigs post-infection.**

| **Group** | **No.** | **qPCR of genomes extracted from lung tissues ^a^** | **Clinical observations** | | | |
| --- | --- | --- | --- | --- | --- | --- |
|  |  | **CT values of lung tissues** | **listless** | **feed intake** | **cough** | **wheezing** |
|  | 1-1 | 22.97 | 19 dpi | \ | \ | \ |
| **168** | 1-2 | 25.74 |  | \ | \ | \ |
|  | 1-3 | 21.64 | 19 dpi | 19 dpi | 19-21 dpi | \ |
|  | 2-1 | 38.38 | \ | \ | \ | \ |
| **168L** | 2-2 | 37.61 | \ | \ | \ | \ |
|  | 2-3 | 37.35 | \ | \ | \ | \ |
|  | 3-1 | 38.75 | \ | \ | \ | \ |
| **XLW-2** | 3-2 | 38.86 | \ | \ | \ | \ |
|  | 3-3 | 37.41 | \ | \ | \ | \ |
|  | 4-1 | 21.50 | 17 dpi | 17 dpi | \ | \ |
| **NJ** | 4-2 | 21.05 | \ | 17 dpi | \ | \ |
|  | 4-3 | 26.13 | \ |  | \ | \ |
|  | 5-1 | 23.01 | \ |  | \ | \ |
| **LH** | 5-2 | 19.04 | \ | 21 dpi | \ | 21-22 dpi |
|  | 5-3 | 22.61 | \ | 21 dpi | \ | \ |
|  | 6-1 | Undet | \ | \ | \ | \ |
| **Control** | 6-2 | Undet | \ | \ | \ | \ |
|  | 6-3 | Undet | \ | \ | \ | \ |

^a^ The presence of *M. hyopneumoniae* in the lungs of these pigs at the end of the experiment was complemented by lung tissue DNA extract and qPCR analysis [[28](#_ENREF_1)].
