# Supplementary material for: Genotyping and biofilm formation of Mycoplasma hyopneumoniae and their association with virulence
Source: Vet Res. 2022 Nov 17;53:95. doi: 10.1186/s13567-022-01109-x (PMC9673451; doi:10.1186/s13567-022-01109-x)
Supplement: Supplementary file 3 — Additional file 3. Correction of virulence and results of the in vitro tracheal infection. [file 13567_2022_1109_MOESM3_ESM.doc]

**Additional file 3. Correction of virulence and results of the in vitro tracheal infection**

The average value of lung lesion and R/G ratio

| **Strains** | **R/G ratio** | **lung lesion score** |
| --- | --- | --- |
| LH | 1.846667 | 12.33333 |
| NJ | 1.766667 | 10.16667 |
| 168 | 1.75 | 10.5 |
| 168L | 1.186667 | 0.333333 |
| XLW-2 | 1.15 | 0 |
| Control | 0.9 | 0 |

**The results of the K-S test for lung lesion and R/G ratio**

|  | **lung lesion** | **R/G ratio** |
| --- | --- | --- |
| **N** | 6 | 6 |
| **Mean±Std.Deviation** | 1.4333±0.40195 | 5.5556±6.01079 |
| **Kolmogorov-Smirnov Z** | 0.285 | 0.308 |
| **Asymp. Sig.(2-tailed)** | 0.140 | 0.079 |

The result of Pearson correlation analysis

|  |  | **OD570nm** | **R/G ratio** |
| --- | --- | --- | --- |
| **lung lesion** | **Pearson Correlation** | 1 | 0.971^**^ |
|  | **Sig. (2-tailed)** |  | .001 |
| **R/G ratio** | **Pearson Correlation** | 0.971^**^ | 1 |
|  | **Sig. (2-tailed)** | .001 |  |

^**^. Correlation is significant at the 0.01 level (2-tailed).
