# Supplementary material for: Genotyping and biofilm formation of Mycoplasma hyopneumoniae and their association with virulence
Source: Vet Res. 2022 Nov 17;53:95. doi: 10.1186/s13567-022-01109-x (PMC9673451; doi:10.1186/s13567-022-01109-x)
Supplement: Supplementary file 4 — Additional file 4. Results of DNA sequencing based on MLST, P146 gene sequencing and MLVA. [file 13567_2022_1109_MOESM4_ESM.docx]

**Additional file 4** **Results of DNA sequencing based on MLST, P146 gene sequencing and MLVA**

| **Typing method** | **Target gene** | **Strains** | **The results of DNA sequencing** |
| --- | --- | --- | --- |
| MLST | *adk* | 168 | GAACTATTTCAAAAATTTTAGTTGAAAAATACAAGTTAGTTCATATTTCTACAGGTGATCTTTTTCGAAAAAAAATTAGTGAAGATTCCCAATTTGCAGCACAAATTCAAAATTATTTAAGTTCAGGAAGTTATGTCCCTGATGAAATAACAAATAAATTAGTTGCTGATTTTATCAAAAAAATCCCTAAAAATCAAGGTTATATTCTTGATGGTTATCCTCGAACGCTTCAACAGTTGGAATTTATGATCAAAAATGGCATCAATCTAGATTGTGTTTTTTATCTAAAAATAAAAAATGAGACGATAATTTCGCGTCTTTCTCAGCGACTTTTTTGTCAAAAATGTCAAAAATCTTATAATTTATTGCTTGCAAAACCGAAAAACGAACTAAAATGTGACCTTGATAACACCGATTTAATTACCAGAAATGATGATCGTCCCGAAATTATCACACATCGAATTGAAAAATTCAATAATTCAGTGATTCCAATTGTTGAATTTTTCAAAAAAAGTGGTATAATATATTATCTAGATGC |
|  |  | 168L | GAACTATTTCAAAAATTTTAGTTGAAAAATACAAGTTAGTTCATATTTCTACAGGTGATCTTTTTCGAAAAAAAATTAGTGAAGATTCCCAATTTGCAGCACAAATTCAAAATTATTTAAGTTCAGGAAGTTATGTCCCTGATGAAATAACAAATAAATTAGTTGCTGATTTTATCAAAAAAATCCCTAAAAATCAAGGTTATATTCTTGATGGTTATCCTCGAACGCTTCAACAGTTGGAATTTATGATCAAAAATGGCATCAATCTAGATTGTGTTTTTTATCTAAAAATAAAAAATGAGACGATAATTTCGCGTCTTTCTCAGCGACTTTTTTGTCAAAAATGTCAAAAATCTTATAATTTATTGCTTGCAAAACCGAAAAACGAACTAAAATGTGACCTTGATAACACCGATTTAATTACCAGAAATGATGATCGTCCCGAAATTATCACACATCGAATTGAAAAATTCAATAATTCAGTGATTCCAATTGTTGAATTTTTCAAAAAAAGTGGTATAATATATTATCTAGATGC |
|  |  | NJ | GAACTATTTCAAAAATTTTAGTTGAAAAATACAAGTTAGTTCATATTTCTACAGGTGATCTTTTTCGAAAAAAAATTAGTGAAGATTCCCAATTTGCAGCACAAATTCAAAATTATTTAAGTTCAGGAAGTTATGTCCCTGATGAAATAACAAATAAATTAGTTGCTGATTTTATCAAAAAAATCCCTAAAAATCAAGGTTATATTCTTGATGGTTATCCTCGAACGCTTCAACAGTTGGAATTTATGATCAAAAATGGCATCAATCTAGATTGTGTTTTTTATCTAAAAATAAAAAATGAGACGATAATTTCGCGTCTTTCTCAGCGACTTTTTTGTCAAAAATGTCAAAAATCTTATAATTTATTGCTTGCAAAACCGAAAAACGAACTAAAATGTGACCTTGATAACACCGATTTAATTACCAGAAATGATGATCGTCCCGAAATTATCACACATCGAATTGAAAAATTCAATAATTCAGTGATTCCAATTGTTGAATTTTTCAAAAAAAGTGGTATAATATATTATCTAGATGC |
|  |  | XLW-2 | GAACTATTTCAAAAATTTTAGTTGAAAAATACAAGTTAGTTCATATTTCTACAGGTGATCTTTTTCGAAAAAAAATTAGTGAAGATTCCCGATTTGCAGCACAAATTCAAAATTATTTAAGTTCAGGAAGTTATGTCCCTGATGAAATAACAAATAAATTAGTTGCTGATTTTATCAAAAAAATCCCTAAAAATCAAGGTTATATTCTTGATGGTTATCCTCGAACGCTTCAACAGTTGGAATTTATGATCAAAAATGGCATCAATCTAGATTGTGTTTTTTATCTAAAAATAAAAAATGAGACGATAATTTCGCGTCTTTCTCAGCGACTTTTTTGTCAAAAATGTCAAAAATCTTATAATTTATTGCTTGCAAAACCGAAAAACGAACTAAAATGTGACCTTGATAACACCGATTTAATTACCAGAAATGATGATCGTCCCGAAATTATCACACATCGAATTGAAAAATTCAATAATTCAGTGATTCCAATTGTTGAATTTTTCAAAAAAAGTGGTATAATATATTATCTAGATGC |
|  |  | RM48 | GAACTATTTCAAAAATTTTAGTTGAAAAATACAAGTTAGTTCATATTTCTACAGGTGATCTTTTTCGAAAAAAAATTAGTGAAGATTCCCAATTTGCAGCACAAATTCAAAATTATTTAAGTTCAGGAAGTTATGTCCCTGATGAAATAACAAATAAATTAGTTGCTGATTTTATCAAAAAAATCCCTAAAAATCAAGGTTATATTCTTGATGGTTATCCTCGAACGCTTCAACAGTTGGAATTTATGATCAAAAATGGCATCAATCTAGATTGTGTTTTTTATCTAAAAATAAAAAATGAGACGATAATTTCGCGTCTTTCTCAGCGACTTTTTTGTCAAAAATGTCAAAAATCTTATAATTTATTGCTTGCAAAACCGAAAAACGAACTAAAATGTGACCTTGATAACACCGATTTAATTACCAGAAATGATGATCGTCCCGAAATTATCACACATCGAATTGAAAAATTCAATAATTCAGTGATTCCAATTGTTGAATTTTTCAAAAAAAGTGGTATAATATATTATCTAGATGC |
|  |  | LH | GAACTATTTCAAAAATTTTAGTTGAAAAATACAAGTTAGTTCATATTTCTACAGGTGATCTTTTTCGAAAAAAAATTAGTGAAGATTCCCAATTTGCAGCACAAATTCAAAATTATTTAAGTTCAGGAAGTTATGTCCCTGATGAAATAACAAATAAATTAGTTGCTGATTTTATCAAAAAAATCCCTAAAAATCAAGGTTATATTCTTGATGGTTATCCTCGAACGCTTCAACAGTTGGAATTTATGATCAAAAATGGCATCAATCTAGATTGTGTTTTTTATCTAAAAATAAAAAATGAGACGGTAATTTCGCGTCTTTCTCAGCGACTTTTTTGTCAAAAATGTCAAAAATCTTATAATTTATTGCTTGCAAAACCGAAAAACGAACTAAAATGTGACCTTGATAACACCGATTTAATTACCAGAAATGATGATCGTCCCGAAATTATCACACATCGAATTGAAAAATTCAATAATTCAGTGATTCCAATTGTTGAATTTTTCAAAAAAAGTGGTATAATATATTATCTAGATGC |
|  |  | J | GAACTATTTCAAAAATTTTAGTTGAAAAATACAAGTTAGTTCATATTTCTACAGGTGATCTTTTTCGAAAAAAAATTAGTGAAGATTCCCAATTTGCAGCACAAATTCAAAATTATTTAAGTTCAGGAAGTTATGTCCCTGATGAAATAACAAATAAATTAGTTGCTGATTTTATCAAAAAAATCCCTAAAAATCAAGGTTATATTCTTGATGGTTATCCTCGAACGCTTCAACAGTTGGAATTTATGATCAAAAATGGCATCAATCTAGATTGTGTTTTTTATCTAAAAATAAAAAATGAGACGATAATTTCGCGTCTTTCTCAGCGACTTTTTTGTCAAAAATGTCAAAAATCTTATAATTTATTGCTTGCAAAACCGAAAAACGAACTAAAATGTGACCTTGATAGCACCGATTTAATTACCAGAAATGATGATCGTCCCGAAATTATCACACATCGAATTGAAAAATTCAATAATTCAGTGATTCCAATTGTTGAATTTTTCAAAAAAAGTGGTATAATATATTATCTAGATGC |
|  | *rpoB* | 168 | AACTTTTACAAAATCAATTTTTGATTGCACTTACAAAAATTGAAAAGAATTCTAAGGAAAAAATTTCAACAAAATCGGATCTTTCGCAGTTAACAGTAAAATCAATTATTAATAATAAGCCAATTTATAATCAATTTAAAAATTTTTTCAACTCTTCAAAACTTTCGCAATTTATGGACCAAATTAACCCGCTTGGCGAAATGGCAAGTAAAAGAAAAGTTACCTCACTTGGCCCTGGCGGTCTAAATCGTGATACTGCTCAATTTGAAGTCCGGGATGTTCATACAACCCATTATGGTAGAATTTGTCCAGTTGAGACTCCCGAGGGGCAAAATATAGGTCTAATTCTTAATTTTTCAGTTTTTTCGCGAATTAATCAATATGGTTTTATTATCACCCCTTATTATCAAGTAAAAAATCGCATTGTTGACTATTCAAAGGTTCACTGGCTAGCCGCATCGGAAGAATTTGATAAAAGTTTTGCACAATCAGGAGTTGAAATCGATCAAAATAATCGAATTATCCCTGATAAATTAACAGTTAGAAAAAATCAAACTTAT |
|  |  | 168L | AACTTTTACAAAATCAATTTTTGATTGCACTTACAAAAATTGAAAAGAATTCTAAGGAAAAAATTTCAACAAAATCGGATCTTTCGCAGTTAACAGTAAAATCAATTATTAATAATAAGCCAATTTATAATCAATTTAAAAATTTTTTCAACTCTTCAAAACTTTCGCAATTTATGGACCAAATTAACCCGCTTGGCGAAATGGCAAGTAAAAGAAAAGTTACCTCACTTGGCCCTGGCGGTCTAAATCGTGATACTGCTCAATTTGAAGTCCGGGATGTTCATACAACCCATTATGGTAGAATTTGTCCAGTTGAGACTCCCGAGGGGCAAAATATAGGTCTAATTCTTAATTTTTCAGTTTTTTCGCGAATTAATCAATATGGTTTTATTATCACCCCTTATTATCAAGTAAAAAATCGCATTGTTGACTATTCAAAGGTTCACTGGCTAGCCGCATCGGAAGAATTTGATAAAAGTTTTGCACAATCAGGAGTTGAAATCGATCAAAATAATCGAATTATCCCTGATAAATTAACAGTTAGAAAAAATCAAACTTAT |
|  |  | NJ | AACTTTTACAAAATCAATTTTTGATTGCACTTACAAAAATTGAAAAGAATTCTAAGGAAAAAATTTCAACAAAATCGGATCTTTCGCAGTTAACAGTAAAATCAATTATTAATAATAAGCCAATTTATAATCAATTTAAAAATTTTTTCAACTCTTCAAAACTTTCGCAATTTATGGACCAAATTAACCCGCTTGGCGAAATGGCAAGTAAAAGAAAAGTTACCTCACTTGGCCCTGGCGGTCTAAATCGTGATACTGCTCAATTTGAAGTCCGGGATGTTCATACAACCCATTATGGTAGAATTTGTCCAGTTGAGACTCCCGAGGGGCAAAATATAGGTCTAATTCTTAATTTTTCAGTTTTTTCGCGAATTAATCAATATGGTTTTATTATCACCCCTTATTATCAAGTAAAAAATCGCATTGTTGACTATTCAAAGGTTCACTGGCTAGCCGCATCGGAAGAATTTGATAAAAGTTTTGCACAATCAGGAGTTGAAATCGATCAAAATAATCGAATTATCCCTGATAAATTAACAGTTAGAAAAAATCAAACTTAT |
|  |  | XLW-2 | AACTTTTACAAAATCAATTTTTGATTGCACTTACAAAAATTGAAAAGAATTCTAAGGAAAAAATTTCAACAAAATCGGATCTTTCGCAGTTAACAGTAAAATCAATTATTAATAATAAGCCAATTTATAATCAATTTAAAAATTTTTTCAACTCTTCAAAACTTTCGCAGTTTATGGACCAAATTAACCCGCTTGGCGAAATGGCAAGTAAAAGAAAAGTTACCTCACTTGGCCCTGGCGGTCTAAATCGTGATACTGCTCAATTTGAAGTCCGGGATGTTCATACAACCCATTATGGTAGAATTTGCCCAGTCGAGACTCCCGAGGGGCAAAATATAGGTCTAATTCTTAATTTTTCAGTTTTTTCGCGAATTAATCAATATGGTTTTATTATCACCCCTTATTATCAAGTAAAAAATCGCATTGTTGACTATTCAAAGGTTCACTGGCTAGCCGCATCGGAAGAATTTGATAAAAGTTTTGCACAATCAGGAGTTGAAATCGATCAAAATAACCGAATTATCCCTGATAAATTAACAGTTAGAAAAAATCAAACTTAT |
|  |  | RM48 | AACTTTTACAAAATCAATTTTTGATTGCACTTACAAAAATTGAAAAGAATTCTAAGGAAAAAATTTCAACAAAATCGGATCTTTCGCAGTTAACAGTAAAATCAATTATTAATAATAAGCCAATTTATAATCAATTTAAAAATTTTTTCAACTCTTCAAAACTTTCGCAATTTATGGACCAAATTAACCCGCTTGGCGAAATGGCAAGTAAAAGAAAAGTTACCTCACTTGGCCCTGGCGGTCTAAATCGTGATACTGCTCAATTTGAAGTCCGGGATGTTCATACAACCCATTATGGTAGAATTTGTCCAGTTGAGACTCCCGAGGGGCAAAATATAGGTCTAATTCTTAATTTTTCAGTTTTTTCGCGAATTAATCAATATGGTTTTATTATCACCCCTTATTATCAAGTAAAAAATCGCATTGTTGACTATTCAAAGGTTCACTGGCTAGCCGCATCGGAAGAATTTGATAAAAGTTTTGCACAATCAGGAGTTGAAATCGATCAAAATAATCGAATTATCCCTGATAAATTAACAGTTAGAAAAAATCAAACTTAT |
|  |  | LH | AACTTTTACAAAATCAATTTTTGATTGCACTTACAAAAATTGAAAAGAATTCTAAGGAAAAAATTTCAACAAAATCGGATCTTTCGCAGTTAACAGTAAAATCAATTATTAATAATAAGCCAATTTATAATCAATTTAAAAATTTTTTTAACTCCTCAAAACTTTCGCAGTTTATGGACCAAATCAACCCGCTTGGGGAAATGGCAAGTAAAAGAAAAGTTACCTCACTCGGCCCTGGCGGTCTAAATCGTGATACTGCTCAATTTGAAGTCCGGGATGTTCATACAACCCATTATGGTAGAATTTGCCCAGTTGAGACTCCCGAGGGGCAAAATATCGGTTTAATTCTTAATTTTTCAGTTTTTTCGCGAATTAATCAATATGGTTTTATTATCACCCCTTATTATCAAGTAAAAAATCGCATTGTTGACTATTCAAAGGTTCACTGGCTAGCCGCATCGGAAGAATTTGATAAAAGTTTTGCACAATCAGGAGTTGAAATTGATCAAAATAACCGAATTATCCCTGATAAATTAACAGTTAGAAAAAATCAAACCTAT |
|  |  | J | AACTTTTACAAAATCAATTTTTGATTGCACTTACAAAAATTGAAAAGAATTCTAAGGAAAAAATTTCAACAAAATCGGATCTTTCGCAGTTAACAGTAAAATCAATTATTAATAATAAGCCAATTTATAATCAATTTAAAAATTTTTTCAACTCCTCAAAACTTTCGCAGTTTATGGACCAAATTAACCCGCTTGGGGAAATGGCAAGTAAAAGAAAAGTTACCTCACTTGGCCCTGGCGGTCTAAATCGTGATACTGCTCAATTTGAAGTCCGGGATGTTCATACAACCCATTATGGTAGAATTTGTCCAGTTGAGACTCCCGAGGGGCAAAATATAGGTCTAATTCTTAATTTTTCAGTTTTTTCGCGAATTAATCAATATGGTTTTATTATCACCCCTTATTATCAAGTAAAAAATCGCATTGTTGACTATTCAAAGGTTCACTGGCTAGCCGCATCGGAAGAATTTGATAAAAGTTTTGCACAATCAGGAGTTGAAATCGATCAAAATAATCGAATTATCCCTGATAAATTAACAGTTAGAAAAAATCAAACTTAT |
|  | *tpiA* | 168 | TGAAACACGTGATTTTATTCAAAAATTTGACATTTTCTATCAGGAAAATGTTGGCAAAATCAAAGAAGATTTAGATTTTGCAATAGCTCCAAGTTTTATATCTTTATCACTAATTTCTAAGTCCTTGACTAAAAAATTAGAAATTGCTGCTCAAAATCTTAGTCAGTTTGATTCAGGAGCCTTTACTGGGGAAATCAGTGGCAAAATGCTGCAGGATTTAGGGACAAAATATGTAATTATTGGGCATTCTGAAAGAAGAGAAATTTTTAAAGAAAAAGATGAAGAACTAAAAAATAAAATTTTACAAGCACAAAAATATGATTTAATTCCTGTTTTTTGTGTTGGTGAAAGTCTTTTAGAATTTGAAGCCGGCCTAACTAAAAAAGTGATAATTTCGCAGATAAATGCTATAAAATCAGTGCTAAATTTTCAAAAGGCAATTATTGCATATGAACCAATTTGGGCCATTGGAACTGGCAAAACAGCAACGGCTGCAATAGCAGAAAAAGTTTGTGGGCTGATTAAGGAAAATTTTGGGAAAAATACAAAGGTAATTTATGGAGGTTCTGTTAATTCTAAGAATATTAA |
|  |  | 168L | TGAAACACGTGATTTTATTCAAAAATTTGACATTTTCTATCAGGAAAATGTTGGCAAAATCAAAGAAGATTTAGATTTTGCAATAGCTCCAAGTTTTATATCTTTATCACTAATTTCTAAGTCCTTGACTAAAAAATTAGAAATTGCTGCTCAAAATCTTAGTCAGTTTGATTCAGGAGCCTTTACTGGGGAAATCAGTGGCAAAATGCTGCAGGATTTAGGGACAAAATATGTAATTATTGGGCATTCTGAAAGAAGAGAAATTTTTAAAGAAAAAGATGAAGAACTAAAAAATAAAATTTTACAAGCACAAAAATATGATTTAATTCCTGTTTTTTGTGTTGGTGAAAGTCTTTTAGAATTTGAAGCCGGCCTAACTAAAAAAGTGATAATTTCGCAGATAAATGCTATAAAATCAGTGCTAAATTTTCAAAAGGCAATTATTGCATATGAACCAATTTGGGCCATTGGAACTGGCAAAACAGCAACGGCTGCAATAGCAGAAAAAGTTTGTGGGCTGATTAAGGAAAATTTTGGGAAAAATACAAAGGTAATTTATGGAGGTTCTGTTAATTCTAAGAATATTAA |
|  |  | NJ | TGAAACACGTGATTTTATTCAAAAATTTGACATTTTCTATCAGGAAAATGTTGGCAAAATCAAAGAAGATTTAGATTTTGCAATAGCTCCAAGTTTTATATCTTTATCACTAATTTCTAAGTCCTTGACTAAAAAATTAGAAATTGCTGCTCAAAATCTTAGTCAGTTTGATTCAGGAGCCTTTACTGGGGAAATCAGTGGCAAAATGCTGCAGGATTTAGGGACAAAATATGTAATTATTGGGCATTCTGAAAGAAGAGAAATTTTTAAAGAAAAAGATGAAGAACTAAAAAATAAAATTTTACAAGCACAAAAATATGATTTAATTCCTGTTTTTTGTGTTGGTGAAAGTCTTTTAGAATTTGAAGCCGGCCTAACTAAAAAAGTGATAATTTCGCAGATAAATGCTATAAAATCAGTGCTAAATTTTCAAAAGGCAATTATTGCATATGAACCAATTTGGGCCATTGGAACTGGCAAAACAGCAACGGCTGCAATAGCAGAAAAAGTTTGTGGGCTGATTAAGGAAAATTTTGGGAAAAATACAAAGGTAATTTATGGAGGTTCTGTTAATTCTAAGAATATTAA |
|  |  | XLW-2 | TGAAACACGTGATTTTATTCAAAAATTTGACATTTTCTATCAGGAAAATGTTGGCAAAATCAAAGAAGATTTAGATTTTGCAATAGCTCCAAGTTTTATATCTTTATCACTAATTTCTAAGTCCTTGACTAAAAAATTAGAAATTGCTGCTCAAAATCTTAGTCAGTTTGATTCAGGAGCCTTTACTGGTGAAATCAGTGGCAAAATGCTGCAGGATTTAGGGACAAAATATGTAATTATTGGGCATTCTGAAAGAAGAGAAATTTTTAAAGAAAAAGATGAAGAACTAAAAAATAAAATTTTACAAGCACAAAAATATGATTTAATTCCTGTTTTTTGTGTTGGTGAAAGTCTTTTAGAATTTGAAGCCGGCCTAACTAAAAAAGTGATAATTTCGCAGATAAATGCTATAAAATCAGTGCTAAATTTTCAAAAGGCAATTATTGCATATGAACCAATTTGGGCCATTGGAACTGGCAAAACAGCAACGGCTGCAATAGCAGAAAAAGTTTGTGGACTGATTAAGGAAAATTTTGGGAAAAATACAATGGTAATTTATGGAGGCTCTGTTAATTCTAAGAATATTAA |
|  |  | RM48 | TGAAACACGTGATTTTATTCAAAAATTTGACATTTTCTATCAGGAAAATGTTGGCAAAATCAAAGAAGATTTAGATTTTGCAATAGCTCCAAGTTTTATATCTTTATCACTAATTTCTAAGTCCTTGACTAAAAAATTAGAAATTGCTGCTCAAAATCTTAGTCAGTTTGATTCAGGAGCCTTTACTGGGGAAATCAGTGGCAAAATGCTGCAGGATTTAGGGACAAAATATGTAATTATTGGGCATTCTGAAAGAAGAGAAATTTTTAAAGAAAAAGATGAAGAACTAAAAAATAAAATTTTACAAGCACAAAAATATGATTTAATTCCTGTTTTTTGTGTTGGTGAAAGTCTTTTAGAATTTGAAGCCGGCCTAACTAAAAAAGTGATAATTTCGCAGATAAATGCTATAAAATCAGTGCTAAATTTTCAAAAGGCAATTATTGCATATGAACCAATTTGGGCCATTGGAACTGGCAAAACAGCAACGGCTGCAATAGCAGAAAAAGTTTGTGGGCTGATTAAGGAAAATTTTGGGAAAAATACAAAGGTAATTTATGGAGGTTCTGTTAATTCTAAGAATATTAA |
|  |  | LH | TGAAACACGTGATTTTATTCAAAAATTTGACATTTTCTATCAGGAAAATGTGGGCAAAATCAAAGAAGATTTAGATTTTGCAATAGCTCCAAGTTTTATATCTTTATCACTAATTTCTAAGTCCTTGACTAAAAAATTAGAAATTGCTGCTCAAAATCTTAGTCAGTTTGATTCAGGAGCCTTTACTGGGGAAATCAGTGGCAAAATGCTGCAGGATTTAGGGACAAAATATGTAATTATTGGGCATTCTGAAAGAAGAGAAATTTTTAAAGAAAAAGATGAAGAACTAAAAAATAAAATTTTACAAGCACAAAAATATGATTTAATTCCTGTTTTTTGTGTTGGTGAAAGTCTTTTAGAATTTGAAGCCGGCCTAACTAAAAAAGTGATAATTTCGCAGATAAATGCTATAAAATCAGTGCTAAATTTTCAAAAGGCAATTATTGCATATGAACCAATTTGGGCCATTGGAACTGGCAAAACAGCAACGGCTGCAATAGCAGAAAAAGTTTGTGGACTGATTAAGGAAAATTTTGGGAAAAATACAATGGTAATTTATGGAGGCTCTGTTAATTCTAAGAATATTAA |
|  |  | J | TGAAACACGTGATTTTATTCAAAAATTTGACATTTTCTATCAGGAAAATGTGGGCAAAATCAAAGAAGATTTAGATTTTGCAATAGCTCCAAGTTTTATATCTTTATCACTAATTTCTAAGTCCTTGACTAAAAAATTAGAAATTGCTGCTCAAAATCTTAGTCAGTTTGATTCAGGAGCCTTTACTGGGGAAATCAGTGGCAAAATGCTGCAGGATTTAGGGACAAAATATGTAATTATTGGGCATTCTGAAAGAAGAGAAATTTTTAAAGAAAAAGATGAAGAACTAAAAAATAAAATTTTACAAGCACAAAAATATGATTTAATTCCTGTTTTTTGTGTTGGTGAAAGTCTTTTAGAATTTGAAGCCGGCCTAACTAAAAAAGTGATAATTTCGCAGATAAATGCTATAAAATCAGTTCTAAATTTTCAAAAGGCAATTATTGCATATGAACCAATTTGGGCCATTGGAACTGGCAAAACAGCAACGGCTGCAATAGCAGAAAAAGTTTGTGGACTGATTAAGGAAAATTTTGGGAAAAATACAATGGTAATTTATGGAGGCTCTGTTAATTCTAAGAATATTAA |
| P146 sequencing | P146 | 168 | AAATACAATTTGAAAAAGAGGCTAATACCACTGAGTCTTCTTCATCTTCTTCATCTTCCTCACCCTCTTCTTCTGAAACCGATACAAATAAACCTGAGAATGCAGTTGAATACAAACTAACTTATTATTATAAAATTTATAATAAAATTACTAAGAAAGTAGTTTATACTACCCCTAAAACAATTAT |
|  |  | 168L | AAATACAATTTGAAAAAGAGGCTAATACCACTGAGTCTTCTTCATCTTCTTCATCTTCCTCACCCTCTTCTTCTGAAACCGATACAAATAAACCTGAGAATGCAGTTGAATACAAACTAACTTATTATTATAAAATTTATAATAAAATTACTAAGAAAGTAGTTTATACTACCCCTAAAACAATTAT |
|  |  | NJ | AAATACAATTTGAAAAAGAGGCTAATACCACTGAGTCTTCTTCATCTTCTTCATCTTCCTCACCCTCTTCTTCTGAAACCGATACAAATAAACCTGAGAATGCAGTTGAATACAAACTAACTTATTATTATAAAATTTATAATAAAATTACTAAGAAAGTAGTTTATACTACCCCTAAAACAATTAT |
|  |  | XLW-2 | AAATACAATTTGAGAAAGAGGCTAATAACACTGAGTCTTCTTCATCCTCTTCATCTTCATCTTCTTCTTCTTCTTCTTCTTCTTCATCTTCTTCTGAAACCGATACAAACAAACCTGAGAATGCAGTTGAATATAAACTAACTTATTATTATAAAATTTATAATAAAACTACTAAGAAAGTAGTTTATACTACCCCTAAAACAATTAT |
|  |  | RM48 | AAATACAATTTGAAAAAGAGGCTAATACCACTGAGTCTTCTTCATCTTCTTCATCTTCCTCACCCTCTTCTTCTGAAACCGATACAAATAAACCTGAGAATGCAGTTGAATACAAACTAACTTATTATTATAAAATTTATAATAAAATTACTAAGAAAGTAGTTTATACTACCCCTAAAACAATTAT |
|  |  | LH | ATAATTGTTTTAGGGGTAGTATAAACTACTTTCTTAGTAGTTTTATTATAAATTTTATAATAATAAGTTAGTTTATATTCAACTGCATTCTCAGGTTTGTTTGTATCGGTTTCAGAAGAAGATGAAGAAGAAGAAGATGAAGATGAAGAGGATGAAGAAGATGAAGAGGATGAAGAGGATGAAGACTCAGTGTTATTAGCCTCTTTCTCAAATTGTATTT |
|  |  | J | AAATACAATTTGAGAAAGAGGCTAATAACACTGAGTCTTCATCCTCTTCATCCTCTTCATCTTCATCTTCTTCTTCTTCATCTTCTTCTGAAACCGATACAAACAAACCTGAGAATGCAGTTGAATATAAACTAACTTATTATTATAAAATTTATAATAAAACTACTAAGAAAGTAGTTTATACTACCCCTAAAACAATTAT |
| MLVA | H2R2 | 168 | TTAATCCAGGCATCATTCTTATCAAAATAAAGATTTACCGATCCTTTGACTTCTTCACCTTCTTCTGGTGCTGAACTACTACTACTACTACTACTACTTTGACTCAATGAACTTCCAGTTCTTACATTAACTTGTTTAACATCGGCAACAAAAATTTTTTGGACTATAGGGGTAAACTCGCTTTTTGTTTGGCTAAGTGATCTTTTACGTCTTGTTCTCACTTGTGTATTCAAATCAGGGATCTCAACCCCACCAATTTCAAGTTTTTTAATTTCATAGGTTTCAAATCTATCAAGATGTTCGAGCCGAAATTCAATTACACCTCTTTTATTTACCGGTTGGAATTTTTCCTTAGAAGTAAATGAAATTTCTTTGTCCTGACTTTTTTCAGAAGATTTTACAACGGTAATTGTTCCATATTCCTTTGGGATTTGTAAAAATCTATCATTAAATGAAAATTTAATTGTTCCTGAACGACCATCAGCATCATCATTTTCATTCTGAGATCAATTTACATCCTCTAGATAATTAAATTTTGCTCTTGTTTCAAAGAAAGGTGCTTTG |
|  |  | 168L | TTAATCCAGGCATCATTCTTATCAAAATAAAGATTTACCGATCCTTTGACTTCTTCACCTTCTTCTGGTGCTGAACTACTACTACTACTACTACTACTTTGACTCAATGAACTTCCAGTTCTTACATTAACTTGTTTAACATCGGCAACAAAAATTTTTTGGACTATAGGGGTAAACTCGCTTTTTGTTTGGCTAAGTGATCTTTTACGTCTTGTTCTCACTTGTGTATTCAAATCAGGGATCTCAACCCCACCAATTTCAAGTTTTTTAATTTCATAGGTTTCAAATCTATCAAGATGTTCGAGCCGAAATTCAATTACACCTCTTTTATTTACCGGTTGGAATTTTTCCTTAGAAGTAAATGAAATTTCTTTGTCCTGACTTTTTTCAGAAGATTTTACAACGGTAATTGTTCCATATTCCTTTGGGATTTGTAAAAATCTATCATTAAATGAAAATTTAATTGTTCCTGAACGACCATCAGCATCATCATTTTCATTCTGAGATCAATTTACATCCTCTAGATAATTAAATTTTGCTCTTGTTTCAAAGAAAGGTGCTTTG |
|  |  | NJ | TTCAATAACAAAGCACCTTTCTTTGAAACAAGAGCAAAATTTAATTATCTAGAGGATGTAAATTGATCTCAGAATGAAAATGATGATGCTGATGGTCGTTCAGGAACAATTAAATTTTCATTTAATGATAGATTTTTACAAATCCCAAAGGAATATGGAACAATTACCGTTGTAAAATCTTCTGAAAAAAGTCAGGACAAAGAAATTTCATTTACTTCTAAGGAAAAATTCCAACCGGTAAATAAAAGAGGTGTAATTGAATTTCGGCTCGAACATCTTGATAGATTTGAAACCTATGAAATTAAAAAACTTGAAATTGGTGGGGTTGAGATCCCTGATTTGAATACACAAGTGAGAACAAGACGTAAAAGATCACTTAGCCAAACAAAAAGCGAGTTTACCCCTATAGTCCAAAAAATTTTTGTTGCCGATGTTAAACAAGTTAATGTAAGAACTGGAAGTTCATTGAGTCAAAGTAGTAGTAGTAGTAGTAGTAGTAGTTCAGCACCAGAAGAAGGTGAAGAAGTCAAAGGATCGGTAAATCTTTATTTTGATAAGAATGATGCCTGGATTAATAGCTCA |
|  |  | XLW-2 | TTCAATAACAAAGCACCTTTCTTTGAAACAAGGGCAAAATTTAATTATCTTGAAGATGTAAACTGATCTCAGGATGAAAATGAAGATGCTGATGGTCGTTCAGGGACAATTAAATTTTCATTTAATGATAGATTTTTACAAATCCCAAAGGAATATGGAACAATTACCGTTGTAAAATCTTCTGAAAAAAATCAGGGCAAAGAAATTTCTTTTACTTCTAAGGAAAAATTCCAACCGGTAAATAAAAAAGGAGTAATTGAATTTCGCCTTGAACATCTTGATAGATTTGAGACCTACGAAATCAAAAAACTTGAAATCGGAGGAGTTGAGATCCCTGATTTGAATACACAAGTGGGAACAAGACGTAAAAGATCACTAAGCCAAACAAAAACCGAGTTTACCCCTATAATCCAAAAAATTTTTGTTGCCGATATAAAACAAGTTAATGTAAGAACAGGAAGTTCATCTAGTCATAGTAGTAGTAGTAGTAGTAGTTCGGGTCCTGAAGAAGGTGAAGAAGTTAAAGGCTTTGTAAATCTTTATTTTGATAAAAATGATGCCTGGATTAATAGCTCA |
|  |  | RM48 | GAGCTTTTAATCCAGGCATCATTCTTATCAAAATAAAGATTTACCGATCCTTTGACTTCTTCACCTTCTTCTGGTGCTGAACTACTACTACTACTACTACTACTTTGACTCAATGAACTTCCAGTTCTTACATTAACTTGTTTAACATCGGCAACAAAAATTTTTTGGACTATAGGGGTAAACTCGCTTTTTGTTTGGCTAAGTGATCTTTTACGTCTTGTTCTCACTTGTGTATTCAAATCAGGGATCTCAACCCCACCAATTTCAAGTTTTTTAATTTCATAGGTTTCAAATCTATCAAGATGTTCGAGCCGAAATTCAATTACACCTCTTTTATTTACCGGTTGGAATTTTTCCTTAGAAGTAAATGAAATTTCTTTGTCCTGACTTTTTTCAGAAGATTTTACAACGGTAATTGTTCCATATTCCTTTGGGATTTGTAAAAATCTATCATTAAATGAAAATTTAATTGTTCCTGAACGACCATCAGCATCATCATTTTCATTCTGAGATCAATTTACATCCTCTAGATAATTAAATTTTGCTCTTGTTTCAAAGAAAGGTGCTTTGTTATTGA |
|  |  | LH | ATAACAAAGCACCTTTCTTTGAAACAAGAGCAAAATTTAATTATCTAGAGGATGTAAATTGATCTCAGAATGAAAATGAAGATGCTGATGGTCGTTCAGGAACAATTAAATTTTCATTTAATGATAGATTTTTACAAATTCCAAAGGAATATGGAACAATTACCGTTGTAAAATCTTCAGAAAAAACTCAGGGCAAAGAAATTTCTTTTACTTCTAAGGAAAAATTCCAACCGGTAAATAAAAAAGGAGTAATTGAATTTCGCCTCGAACATCTTGATAGATTTGAGACCTATGAAATCAAAAAACTTGAAATCGGAGGAGTTGAGATCCCTGATTTGAATACACAAGTGGGAACAAGACGTAAAAGATCACTTAGCCAAACAAAAAGTGAATTTACCCCTATAATCCAAAAAATTTTTGTTGCCGATGTTAAACAAGTTAATGTAAGAACTGGAAGTTCATCGAGTCAAAGTAGTAGTAGTAGTAGTAGTAGTAGTAGTTCAGCACCAGAAGAAGGTGAAAACGTCA |
|  |  | J | GAGCTATTAATCCAGGCATCATTCTTATCAAAATAAAGATTTACATACCCTTTTACTTCTTCACCTTCTTCTGGACCTGAACTACTACTACTACTACTACTACTACTACTACTATGACTCGATGAACTTCCTGTTCTTACATTAACTTGTTTAACATCGGCAACAAAAATTTTCTGGATTATAGGGGTAAACTCGCTTTTTGTTTGGCTAAGTGATCTTTTACGTCTTGTTCTCACTTGTGTATTCAAATCAGGGATCTCAACCCCACCAATTTCAAGTTTTTTAATTTCATAGGTCTCAAATCTATCAAGATGTTCAAGACGAAATTCAATTACTCCCTTTTTATTTACCGGCTGGAATTTTTCCTTAGAAGTAAATGAAATTTCTTTTCCATTAGAGTTTGCCTCTGAAGATTTTACAACGGTAATTGTTCCATATTCCTTTGGGATTTGTAAAAATCTATCATTAAATGAAAATTTAATTGTTCCTGAACGACCATCGGCATCTTCATTTTCATCCTGAGATCAATTTACATCTTCAAGATAATTAAATTTTGCTCTTGTTTCAAAGAAAGGTGCTTTGTTATTGA |
|  | H3 | 168 | GTTAAAAAAGCGGCTTTTCCGGAATTAGCGTTTAAAAAAACTGCAGTTCCAGGGGCAAACCGAATGTTTCGAGTAAAAATATTTGCATATCTTTTTTCTTCAAGACTATCTGATTTAAATCCAAAGTAAGGTAGAATATAATAATTTCCTAGTCTATTTGGGTTAAAATAGGAATAATAATTTGCTTTTGCATTAACATTATCGACTAAATTATTATTCATTTTTTTTATTAGAAAAATATTTAAATTATCATTTTTCTGACCTAAAAAAGTAGTTTCATTTTCTTTTTTTATAACATTTTCCCAATTTTTAAGCTGATATTTATAATAAGTTGGCGTTTTAGCAATATTTTTTACCTCATTTTTGCTAAAATCGCTTTCGCTGGCTTGTGTTAGTTCATTAACATTAATTTTTATTGAATTTTGATTTACCTTAGCAAAATCAAAACTAGTTTGGTCTGATAAATCACTTGTAGATTCGGACCAAAAATTGCTATCAGTGGTTTGATTATTTTCCGTTCTTTCTGTTTTTTCTGTTTTATTTTGGCTATTTACTACATCTTTTTTTTGGGTATTTTCTTCAACTGGACTTACTTTTGATTCTTTTGTTGGATCTAATTTATCTTCACTTTTTTCGGGAGTTTCTTCACTTCTTATT |
|  |  | 168L | AAAAAAGCGGCTTTTCCGGAATTAGCGTTTAAAAAAACTGCAGTTCCAGGGGCAAACCGAATGTTTCGAGTAAAAATATTTGCATATCTTTTTTCTTCAAGACTATCTGATTTAAATCCAAAGTAAGGTAGAATATAATAATTTCCTAGTCTATTTGGGTTAAAATAGGAATAATAATTTGCTTTTGCATTAACATTATCGACTAAATTATTATTCATTTTTTTTATTAGAAAAATATTTAAATTATCATTTTTCTGACCTAAAAAAGTAGTTTCATTTTCTTTTTTTATAACATTTTCCCAATTTTTAAGCTGATATTTATAATAAGTTGGCGTTTTAGCAATATTTTTTACCTCATTTTTGCTAAAATCGCTTTCGCTGGCTTGTGTTAGTTCATTAACATTAATTTTTATTGAATTTTGATTTACCTTAGCAAAATCAAAACTAGTTTGGTCTGATAAATCACTTGTAGATTCGGACCAAAAATTGCTATCAGTGGTTTGATTATTTTCCGTTCTTTCTGTTTTTTCTGTTTTATTTTGGCTATTTACTACATCTTTTTTTTGGGTATTTTCTTCAACTGGACTTACTTTTGATTCTTTTGTTGGATCTAATTTATCTTCACTTTTTTCGGGAGTTTCTTCACTTCTTATT |
|  |  | NJ | AAAAGCGGCTTTTCCGGAATTAGCGTTTAAAAAAACTGCAGTTCCAGGGGCAAACCGAATGTTTCGAGTAAAAATATTTGCATATCTTTTTTCTTCAAGACTATCTGATTTAAATCCAAAGTAAGGTAGAATATAATAATTTCCTAGTCTATTTGGGTTAAAATAGGAATAATAATTTGCTTTTGCATTAACATTATCGACTAAATTATTATTCATTTTTTTTATTAGAAAAATATTTAAATTATCATTTTTCTGACCTAAAAAAGTAGTTTCATTTTCTTTTTTTATAACATTTTCCCAATTTTTAAGCTGATATTTATAATAAGTTGGCGTTTTAGCAATATTTTTTACCTCATTTTTGCTAAAATCGCTTTCGCTGGCTTGTGTTAGTTCATTAACATTAATTTTTATTGAATTTTGATTTACCTTAGCAAAATCAAAACTAGTTTGGTCTGATAAATCACTTGTAGATTCGGACCAAAAATTGCTATCAGTGGTTTGATTATTTTCCGTTCTTTCTGTTTTTTCTGTTTTATTTTGGCTATTTACTACATCTTTTTTTTGGGTATTTTCTTCAACTGGACTTACTTTTGATTCTTTTGTTGGATCTAATTTATCTTCACTTTTTTCGGGAGTTTCTTCACTTCTTATTTCC |
|  |  | XLW-2 | CGGCTTTTCCGGAATTGGCGTTTAAAAAAACTGCAGTTCCAGGGGCAAACCGAATGTTTCGAGTAAAAATATTTGCATATCTTTTTTCTTCAAGACTATCTGATTTAAATCCAAAGTAAGGTAGACTATAATAATCTCCTAGTTTATTTGCTGGGTTAAAATAGGAATAATAATTTGCTTTTGCATTAACATTATCGACTAAATTATTATTCATTTGTTTTATTAGAAAAATATTTAAATTATCATTTTTCTGACCTAAAAAAGTAGTTTCATTTTCCTTTTTTATAACATTTTCCCAATTTTTAAGCTGATATTTATAATAAGTTGGCGTTTTAGCAATATTTTTTACCTCATTTTTGCTAAAATCGCTTTCGCTGGCCTGTGTTAGTCCATTAATATTAATTTTTATTGAATTTTGATTTACCTTAGTAAAATCAAAACTAGTTTGGTCTGATAAATCACTTGTAGAGTCAGACCAAAAATTGTTATCAGTGGTTTGATTTTCCGTTCTTTCTGTTTTTTCTGTTTTTTCTGTTTTTTCTGTTTTTTCTGTTTTATTTTGGCTATTTACTACATCTTTTTTTTGGGTATTTTTTTCAACTGGACTTACTTTTGATTCTTTTGTTGGATCTAATTTATCTTCACTTTTTTCGGGAGTTTCTTCACTTCTTATT |
|  |  | RM48 | AAAAAAGCGGCTTTTCCGGAATTAGCGTTTAAAAAAACTGCAGTTCCAGGGGCAAACCGAATGTTTCGAGTAAAAATATTTGCATATCTTTTTTCTTCAAGACTATCTGATTTAAATCCAAAGTAAGGTAGAATATAATAATTTCCTAGTCTATTTGGGTTAAAATAGGAATAATAATTTGCTTTTGCATTAACATTATCGACTAAATTATTATTCATTTTTTTTATTAGAAAAATATTTAAATTATCATTTTTCTGACCTAAAAAAGTAGTTTCATTTTCTTTTTTTATAACATTTTCCCAATTTTTAAGCTGATATTTATAATAAGTTGGCGTTTTAGCAATATTTTTTACCTCATTTTTGCTAAAATCGCTTTCGCTGGCTTGTGTTAGTTCATTAACATTAATTTTTATTGAATTTTGATTTACCTTAGCAAAATCAAAACTAGTTTGGTCTGATAAATCACTTGTAGATTCGGACCAAAAATTGCTATCAGTGGTTTGATTATTTTCCGTTCTTTCTGTTTTTTCTGTTTTATTTTGGCTATTTACTACATCTTTTTTTTGGGTATTTTCTTCAACTGGACTTACTTTTGATTCTTTTGTTGGATCTAATTTATCTTCACTTTTTTCGGGAGTTTCTTCACTTCTTATT |
|  |  | LH | TGTGTTAAAAAAGCGGCTTTTCCGGAATTAGCGTTTAAAAAAACTGCAGTTCCAGGGGCAAACCGAATGTTTCGAGTAAAAATATTTGCATATCTTTTTTCTTCAAGACTATCTGATTTAAATCCAAAGTAAGGTAGAATATAATAATTTCCTAGTCTATTTGGGTTAAAATAGGAATAATAATTTGCTTTTGCATTAACGTTATCGACTAAATTATTATTCATTTGTTTTATTAGAAAAATATTTAAATTATCATTTTTCTGACCTAAAAAAGTAGTTTCATTTTCTTTTTTTATAACATTTTCCCAATTTTTAAGCTGATATTTATAATAAGTTGGCGTTTTAGCAATATTTTTTACCTCATTTTTGCTAAAATCGCTTTCGCTGGCTTGTGTTAGTCCATTAATATTAATTTTTATTGAATTTTGATTTACCTTTGCAAAATCAAAATTAGTTTGGTCTGATAAATCACTTGTAGATTCAGACCAAAAATTGTTATCTGTGGTTTGATTTTCCGTTCCTTGTGTTTTTTCCGTTTTTTCTGTTTTATTTTGGCTATTTACTACATCATTTTTTTGGCTATTTTCTTTTTGGGTATTTTCTTCAACTGGACTTACTTTTGATTCTTTTGTTGGATCTAATTTATCTTCACTTTTTTCGGGAGTTTCTTCACTTCTTATTTCCGGTAAT |
|  |  | J | CGGCTTTTCCGGAATTAGCGTTTAAAAAAACTGCAGTTCCAGGGGCAAACCGAATGTTTCGAGTAAAAATATTTGCATATCTTTTTTCTTCAAGACTATCTGATTTAAATCCAAAGTAAGGTAGAATATAATAATTTCCTAGTCTATTTGGGTTAAAATAGGAATAATAATTTGCTTTTGCATTAACATTATCGACTAAATTATTATTCATTTTTTTTATTAGAAAAATATTTAAATTATCATTTTTCTGACCTAAAAAAGTAGTTTCATTTTCTTTTTTTATAACATTTTCCCAATTTTTAAGCTGATATTTATAATAAGTTGGCGTTTTAGCAATATTTTTTACCTCATTTTTGCTAAAATCGCTTTCGCTGGCTTGTGTTAGTTCATTAACATTAATTTTTGTTGAATTTGGATTTACCTTAGCAAAATCAAAACTAGTTTGGTCTGATAAATCACTTGTAGATTCGGACCAAAAATTGCTATCAGTGGTTTGATTATTTTCCGTTCTTTCTGTTTTTTCTGTTTTATTTTGGCTATTTACTACATCTTTTTTTTGGGTATTTTCTTCAACTGGACTTACTTTTGATTCTTTTGTTGGATCTAATTTATCTTCACTTTTTTCGGGAGTTTCTTCACTTCTTATTTCCGGTAAT |
|  | H5R1/ R2 | 168 | AGTAAAAAAGACCTCGAAGAATATAAAGAGAAACACAAAAACAAGTTTATTAACGAAATAAAACCTGCTACACCAACAAG  TCAAGCAAAAACAAGTCAAGCAAAAACAAGTCAAGCAAAAAATGAAAAAGAAGTAAAACCTGAATCAGCCCAAGCAGAATCTTCATCTTCAAATTCTAGTGATTCTAATAGTAAAACCACTTCTTCTTCAAGTATGGCGGGTACAACCCAAAATAAATCT  ACAGAAACTACAAATTCAAGTTCAAATTCAACACCAACAAGTTCAACAACAAGTTCAACAACAAGTTCAACAACACAAGC  AGCAACAACTTCAGCCTCTTCGGCTAAAGTAAAAACAACTAAATTCCAAGAACAAGTAAAAGAACAAGAACAAGAAAAAGGAAAAGAAACTAACCAATTATTAGATAGTAGTAAAACAAATAAAGAAAACTTAGGACTTGGATTAATTCTTTGG |
|  |  | 168L | AAGTAAAAAAGACCTCGAAGAATATAAAGAGAAACACAAAAACAAGTTTATTAACGAAATAAAACCTGCTACACCAACAA  GTCAAGCAAAAACAAGTCAAGCAAAAACAAGTCAAGCAAAAAATGAAAAAGAAGTAAAACCTGAATCAGCCCAAGCAGAATCTTCATCTTCAAATTCTAGTGATTCTAATAGTAAAACCACTTCTTCTTCAAGTATGGCGGGTACAACCCAAAATAAATC  TACAGAAACTACAAATTCAAGTTCAAATTCAACACCAACAAGTTCAACAACAAGTTCAACAACAAGTTCAACAACACAAG  CAGCAACAACTTCAGCCTCTTCGGCTAAAGTAAAAACAACTAAATTCCAAGAACAAGTAAAAGAACAAGAACAAGAAAAAGGAAAAGAAACTAACCAATTATTAGATAGTAGTAAAACAAATAAAGAAAACTTAGGACTTGGATTAATTCTTTGG |
|  |  | NJ | CCTCGAAGAATATAAAGAGAAACACAAAAACAAGTTTATTAACGAAATAAAACCTGCTACACCAACAAGTCAAGCAAAAACAAGTCAAGCAAAAACAAGTCAAGCAAAAAATGAAAAAGAAGTAAAACCTGAATCAGCCCAAGCAGAATCTTCATCTTCAAATTCTAGTGATTCTAATAGTAAAACCACTTCTTCTTCAAGTATGGCGGGTACAACCCAAAATAAATCTACAGAAACTACAAATTCAAGTTCAAATTCAACACCAACAAGTTCAACAACAAGTTCAACAACAAGTTCAACAACACAAGCAGCAACAACTTCAGCCTCTTCGGCTAAAGTAAAAACAACTAAATTCCAAGAACAAGTAAAAGAACAAGAACAAGAAAAAGGAAAAGAAACTAACCAATTATTAGATAGTAGTAAAACAAATAAAGAAAACTTAGGACTTGGAT |
|  |  | XLW-2 | AGTAAAAAAGACCTCGAAGAATATAAAGAAAAACACAAAAACAAGTTTATCAGCGAAATAAAACCTGCTACACCAGCAAGTCAAGCAAAAACAAGTCAAGCAAAAAATGAAAAAGAAGTAGAACCTGAATCAGCCCAAGCAGAATCTTCATCTTCAAATTCTAGTGATTCTAGTAGTAAAACCACTTCTTCTTCTTCAAGTATGATGGCGGGTACAACCCAAACAAATAATTCCTCTACAGAAACAACAAATTCAAATTCAGCAACAACAAGTTCAACAACCTCAACACAAGCAGCAACAACTTCAGCCTCTTCGGCTAAAGTTAAAACAACTAAATTCCAAGAACAAGTAAAAGAACAAAAACAAAAACAAGAAAAAACAAAAGAAACTAACCAATTATTAAATAGTAAAAGAAATAAAGAAGACTCTGGACTTGGATTAATTCTTTGGA |
|  |  | RM48 | CCTCGAAGAATATAAAGAGAAACACAAAAACAAGTTTATTAACGAAATAAAACCTGCTACACCAACAAGTCAAGCAAAAACAAGTCAAGCAAAAACAAGTCAAGCAAAAAATGAAAAAGAAGTAAAACCTGAATCAGCCCAAGCAGAATCTTCATCTTCAAATTCTAGTGATTCTAATAGTAAAACCACTTCTTCTTCAAGTATGGCGGGTACAACCCAAAATAAATCTACAGAAACTACAAATTCAAGTTCAAATTCAACACCAACAAGTTCAACAACAAGTTCAACAACAAGTTCAACAACACAAGCAGCAACAACTTCAGCCTCTTCGGCTAAAGTAAAAACAACTAAATTCCAAGAACAAGTAAAAGAACAAGAACAAGAAAAAGGAAAAGAAACTAACCAATTATTAGATAGTAGTAAAACAAATAAAGAAAACTTAGGACTTGGATTAATTCTTTGG |
|  |  | LH | GTAAAAAAGACCTCGAAGAATATAAAGAAAAACACAAAAACAAGTTTATCAGCGAAATAAAACCTGCTACACCAGCAAGTCAAGCAAAAACAAGTCAAGCAAAAAATGAAAAAGAAGTAAAACCTGAATCAGCCCAAGCAGAAGTTTCATCTTCAAATTCTAATGATTCTAATAGTAAAACCACTTCTTCTTCTTCTTCAAGTATGATGGCGGGTACAACCCAAACAAATAATTCCTCTACAGAAACAACAAATTCAAATTCAGCAACAACAAGTTCAACAACTTCAACACAAGCAGCAACAACTTCAGCCTCTTCGGCTAAAGTTAAAACAACTAAATTCCAAGAACAAGTAAAAGAACAAGAACAAAAACAAGAAAAAGCAAAAGAAACTAACCAATTATTAAATAGTAAAAGAAATAAAGAAGACTCTGGACTTGGATTAATTCTTTGGA |
|  |  | J | CTCGAAGAATATAAAGAGAAACACAAAAACAAGTTTATTAACGAAATAAAACCTGCTACAGCAACAAGTCAAGCAAAACCAGATCAAGCAAAAAATGAAAAAGAAGTAAAACCTGAATCAGCCCAAGCAGAATCTTCATCTTCAAATTCTAATGATTCTAATAGTAAAACCACTTCTTCTTCAAGTATGGCGGGTACAACCCAAAATAAATCTACAGAAACTCCAAATTCAAGTTCAAATTCAACACCAACAAGTTCAGCAACAACTTCAACAACAAGTTCAACACAAGCAGCAGCAACTTCAGCCTCTTCGGCTAAAGTAAAAACAACTAAATTCCAAGAGCAAGAAAAACAACAAGTAAAAGAACAAAAACAAAAACAAGAAAAAACAAAAGAAACTAACCAATTATTAGATACTAAAACAAATAAAGAAAACTTAGGACTTGGAT |
|  | H6R3 | 168 | TCCAGGGGCAAAATATCAAGTAATCGGCCTTTTAGATCCCCAACAAAAAATTAGAATAAATGTACCTAATGAAAATGGG  ATTACTACTTCACTTTTTTCTTCTTCTTCTACAAGCCAACAAAATACTCCTTCATTTAATTTTAATACGGCCCCTTTAAT  TACCAAACTAGCTTATGTAGCCCGTGAAAATTCAATAAAGTTAATTTTTGATGTAGAAAACTCACAAAAATTAACTTTTG  AAAATTCAAAAATGGAGATAAAATATAAAAAATTAAAAAATAAATATCAAGAATATGGCTGACAAAACCCTAGTGATCCT  ATTGATAAAACGCCTAAGACTTCTTCTGAACTCCAAGTGAGTGCAAAAGAAGTAAAAAACGAACAAATACTAATAACAAA  GTCAAAAAATGATGATTCAACCCTAACTCGTCTTGAA |
|  |  | 168L | TCCAGGGGCAAAATATCAAGTAATCGGCCTTTTAGATCCCCAACAAAAAATTAGAATAAATGTACCTAATGAAAATGGG  ATTACTACTTCACTTTTTTCTTCTTCTTCTACAAGCCAACAAAATACTCCTTCATTTAATTTTAATACGGCCCCTTTAAT  TACCAAACTAGCTTATGTAGCCCGTGAAAATTCAATAAAGTTAATTTTTGATGTAGAAAACTCACAAAAATTAACTTTTG  AAAATTCAAAAATGGAGATAAAATATAAAAAATTAAAAAATAAATATCAAGAATATGGCTGACAAAACCCTAGTGATCCT  ATTGATAAAACGCCTAAGACTTCTTCTGAACTCCAAGTGAGTGCAAAAGAAGTAAAAAACGAACAAATACTAATAACAAA  GTCAAAAAATGATGATTCAACCCTAACTCGTCTTGAA |
|  |  | NJ | TCCAGGGGCAAAATATCAAGTAATCGGCCTTTTAGATCCCCAACAAAAAATTAGAATAAATGTACCTAATGAAAATGGG  ATTACTACTTCACTTTTTTCTTCTTCTTCTACAAGCCAACAAAATACTCCTTCATTTAATTTTAATACGGCCCCTTTAAT  TACCAAACTAGCTTATGTAGCCCGTGAAAATTCAATAAAGTTAATTTTTGATGTAGAAAACTCACAAAAATTAACTTTTG  AAAATTCAAAAATGGAGATAAAATATAAAAAATTAAAAAATAAATATCAAGAATATGGCTGACAAAACCCTAGTGATCCT  ATTGATAAAACGCCTAAGACTTCTTCTGAACTCCAAGTGAGTGCAAAAGAAGTAAAAAACGAACAAATACTAATAACAAA  GTCAAAAAATGATGATTCAACCCTAACTCGTCTTGAA |
|  |  | XLW-2 | TTCCAGGGGCAAAATATCAAGTAATCGGCCTTTTAGATCCCCAACAAAAAATTAGAATAAATGTACCTAATGAAAATGGG  ATTACTACTTCACTTTTTTCTTCTTCTTCTACAAGCCAACAAAATACTCCTTCATTTAATTTTAATACGGCCCCTTTAAT  TACCAAACTAGCTTATGTAGCCCGTGAAAATTCAATAAAGTTAATTTTTGATGTAGAAAACTCACAAAAATTAACTTTTG  AAAATTCAAAAATGGAGATAAAATATAAAAAATTAAAAAATAAATATCAAGAATATGGCTGACAAAACCCTAGTGATCCT  ATTGATAAAACGCCTAAGACTTCTTCTGAACTCCAAGTGAGTGCAAAAGAAGTAAAAAACGAACAAATACTAATAACAAA  GTCAAAAAATGATGATTCAACCCTAACTCGTCTTGAA |
|  |  | RM48 | TCCAGGGGCAAAATATCAAGTAATCGGCCTTTTAGATCCCCAACAAAAAATTAGAATAAATGTACCTAATGAAAATGGG  ATTACTACTTCACTTTTTTCTTCTTCTTCTACAAGCCAACAAAATACTCCTTCATTTAATTTTAATACGGCCCCTTTAAT  TACCAAACTAGCTTATGTAGCCCGTGAAAATTCAATAAAGTTAATTTTTGATGTAGAAAACTCACAAAAATTAACTTTTG  AAAATTCAAAAATGGAGATAAAATATAAAAAATTAAAAAATAAATATCAAGAATATGGCTGACAAAACCCTAGTGATCCT  ATTGATAAAACGCCTAAGACTTCTTCTGAACTCCAAGTGAGTGCAAAAGAAGTAAAAAACGAACAAATACTAATAACAAA  GTCAAAAAATGATGATTCAACCCTAACTCGTCTTGAA |
|  |  | LH | TCAGATCAAATGGCTGTAACAAAAAATGGAGGCAGTTCCCAAAATCAAAATAGGAAAGCTGAGCTCCGTCAAGGTTGATTTGAAGTTGAATTTGCCAAAAAAGATCTAGGATTTTTAAAAGATAAATATAAAATTCAGCTTGAGCTTGAATCAGTTGATAAAGATGTTTTTTATACAAATACAGTCGAAATTAAAGATAGTAGTAGTAGTAGTAGTAGTAGTAATACAATCAAAGTCCAACTGGAAGCTAAAAATTTAACTCCTGGGGATAGATATACAATCAAGAATTATATTTTTACTTTAAAACCAGAAATGGCCAACAAATTTGCCGTTAGCCTGCCGCAGAATTTAAGGGCAAAACCAAAAAGAACTAATTATAACCTTTTAACCGAAAATGCAATCAAATCAATTAAATATGAAGCAATCCGTGAAGGA |
|  |  | J | CCAGGGGCAAAGTATCAAGTAATCGGGCTTTTAGATCCCCAACAAAAAATTAGAATAAATGTACCTAATGAAAATGGGAT  TACTACTTCACTTTTTTCTTCTTCAAGCCCACAAAATACTCCTTCATTTAATTTTAACACGGCCCCTTTAATTACCAAAC  TAGCTTATGTAGCCCGCGAGAATTCAATAAAGTTAATTTTTGATGTTGAAAACTCACAAAAATTAACTTTTGAAAATTCA  AAAATGGAAATAAAATATAAAAAATTAAAAAATAAATATCAAGAATATGGCTGGCAAAACCCTAGTGATCCTATTGATAA  AATGGCTAAGACTTCTTCTGAACTCCAAGTTAGTGCAAAAGAGGTAAAAAACGAACAAATACTAATAACAAAGTCAAAAA  ATGATGATTCAACCCTAA |
|  | CH | 168 | GTGAATGTCAAATCTTCTAAGGCAGAAAAAGATCAAGAAAATAAAATAAATGAATCAATTAAAAAAGTGACTAGAAAAATAAATTCCACTTTAAGAAAAACAAAAAATGATAATCAAAGTGATATTCTAATAGAAAAATCACCAAATTCTAATCAGAAAAACCCAAATAAATTTAAAAATAAAAATCCTCAAAAAACCGAAAACTTAGTATTCTTTAAAAAAGAAAATTTAAAAAAAAGCTATGAAAATGATGCTAAAAAAAAGATAAAAAAAAGTCTTATAAACTCAAAAAATTTACCTTATAATAATAATAATAAAGAAAATGTTAAAAATTCAAGAGATCCACAAGCCTTATTAGCCAGAAAAAAACTTTATCCTTTAAAAAATAAGAAGCAAAATACCCTTTTTCAAACGAAAAAACAAGAACTGAATATACCAAAA |
|  |  | 168L | GGCAAAAAAATGTGAATGTCAAATCTTCTAAGGCAGAAAAAGATCAAGAAAATAAAATAAATGAATCAATTAAAAAAGT  GACTAGAAAAATAAATTCCACTTTAAGAAAAACAAAAAATGATAATCAAAGTGATATTCTAATAGAAAAATCACCAAATT  CTAATCAGAAAAACCCAAATAAATTTAAAAATAAAAATCCTCAAAAAACCGAAAACTTAGTATTCTTTAAAAAAGAAAAT  TTAAAAAAAAGCTATGAAAATGATGCTAAAAAAAAGATAAAAAAAAGTCTTATAAACTCAAAAAATTTACCTTATAATAA  TAATAATAAAGAAAATGTTAAAAATTCAAGAGATCCACAAGCCTTATTAGCCAGAAAAAAACTTTATCCTTTAAAAAATA  AGAAGCAAAATACCCTTTTTCAAACGAAAAAACAAGAACTGAATAT |
|  |  | NJ | AAAAAAATGTGAATGTCAAATCTTCTAAGGCAGAAAAAGATCAAGAAAATAAAATAAATGAATCAATTAAAAAAGTGACTAGAAAAATAAATTCCACTTTAAGAAAAACAAAAAATGATAATCAAAGTGATATTCTAATAGAAAAATCACCAAATTCTAATCAGAAAAACCCAAATAAATTTAAAAATAAAAATCCTCAAAAAACCGAAAACTTAGTATTCTTTAAAAAAGAAAATTTAAAAAAAAGCTATGAAAATGATGCTAAAAAAAAGATAAAAAAAAGTCTTATAAACTCAAAAAATTTACCTTATAATAATAATAATAAAGAAAATGTTAAAAATTCAAGAGATCCACAAGCCTTATTAGCCAGAAAAAAACTTTATCCTTTAAAAAATAAGAAGCAAAATACCCTTTTTCAAACGAAAAAACAAGAACTGAATATACCAAAAA |
|  |  | XLW-2 | GTGAATGTCAAATCTTCTAAAACAGAAAAAGATCAAGAAAATAAAATAAAAAAATCAATTGAAAAAGTGACTAGAAAAATAAATTCCACTTTAAGAAAAACAAAAAATGATAATCAAAGTGATATTCTAATAGAAAAATCACCAAATTCTAATCAGAAAAACCCAAATAAATTTAAAAATAAAAATCCTCAAAAAACCGAAAACTTAGTATTCTTTAAAAAAGAAAATTTAAAAAAAAGCTATGAAAATGATGCTAAAAAGATAAAAAAAAGTCTTATAAACTCAAAAAATTTACCTTATAATAATAATAATAATAAAGAAAATGTTAAAAATTCAGGAGATCCACAAGCCTTATTAGCCAGAAAAAAACTTTATCCTTTAAAAAATAAGAAACAAAATACCCTTTTTCAAACGAAAAAACAAGAACTGAATATACCAAAAAG |
|  |  | RM48 | AAAAAAATGTGAATGTCAAATCTTCTAAGGCAGAAAAAGATCAAGAAAATAAAATAAATGAATCAATTAAAAAAGTGACTAGAAAAATAAATTCCACTTTAAGAAAAACAAAAAATGATAATCAAAGTGATATTCTAATAGAAAAATCACCAAATTCTAATCAGAAAAACCCAAATAAATTTAAAAATAAAAATCCTCAAAAAACCGAAAACTTAGTATTCTTTAAAAAAGAAAATTTAAAAAAAAGCTATGAAAATGATGCTAAAAAAAAGATAAAAAAAAGTCTTATAAACTCAAAAAATTTACCTTATAATAATAATAATAAAGAAAATGTTAAAAATTCAAGAGATCCACAAGCCTTATTAGCCAGAAAAAAACTTTATCCTTTAAAAAATAAGAAGCAAAATACCCTTTTTCAAACGAAAAAACAAGAACTGAAT |
|  |  | LH | TGTGAATGTCAAATCTTCTAAAACAGAAAAAGATCAAGAAAATAAAATAAAAAAATCAATTGAAAAAGTGACTAGAAAAATAAATTCCACTTTAAGAAAAACAAAAAATGATAATCAAAGTGATATTCTAATAGAAAAATCACCAAATTCTAATCAGAAAAACCCAAATAAATTTAAAAATAAAAATCCTCAAAAAACCGAAAACTTAGTATTCTTTAAAAAAGAAAATTTAAAAAAAAGCTATGAAAATGATGCTAAAAAGATAAAAAAAAGTCTTATAAACTCAAAAAATTTACCTTATAATAATAATAATAATAATAAAGAAAATGTTAAAAATTCAGGAGATCCACAAGCCTTATTAGCCAGAAAAAAACTTTATCCTTTAAAAAATAAGAAACAAAATACCCTTTTTCAAACGAAAAAACAAGAACTGAATATAC |
|  |  | J | GAATGTCAAATCTTCTAAGGCAGAAAAAGATCAAGAAAATAAAATAAATGAATCAATTAAAAAAGTGACTAGAAAAATAAATTCCACTTTAAGAAAAACAAAAAATGATAATCAAAGTGATATTCTAATAGAAAAATCACCAAATTCTAATCAGAAAAACCCAAATAAATTTAAAAATAAAAATCCTCAAGAAACCGAAAACTTAGTATTCTTTAAAAAAGAAAATTTAAAAAAAAGCTATGAAAATGATGCTAAAAAAAAGATAAAAAAAAGTCTTATAAACTCAAAAAATTTACCTTATAATAATAAAGAAAATGTTAAAAATTCAAGAGATCCACAAGCCTTATTAGCCAGAAAAAAACTTTATCCTTTAAAAAATAAGAAGCAAAATACCCTTTTTCAAACGAAAAAACAAGAACTGAATATACCAAAAAG |
|  | P97R1 | 168 | GAAGCTATCAAAAAAGGGGAAACTACAAAAGAAGGTAAAAGAGAAGAAGTAGATAAAAAAGTTAAAGAATTAGATAATAAAATAAAAGGTATATTACCTCAGCCCCCAGCAGCTAAACCTGAAGCAGCAAAACCAGTAGCAGCTAAACCTGAAGCAGCAAAACCAGTAGCAGCTAAACCTGAAGCAGCAAAACCAGTAGCAGCTAAACCTGAAGCAGCAAAACCAGTAGCGGCTAAACCTGAAGCAGCTAAACCAGTAGCAGCTAAACCAGTTGCTACTAATACTAATACTAATACTGGCTTTTCACTTACAAATAAACC |
|  |  | 168L | GGTTTATTTGTAAGTGAAAAGCCAGTATTAGTATTAGTATTAGTAGCAACTGGTTTAGCTGCTACTGGTTTAGCTGCTTCAGGTTTAGCCGCTACTGGTTTTGCTGCTTCAGGTTTAGCTGCTACTGGTTTTGCTGCTTCAGGTTTAGCTGCTACTGGTTTTGCTGCTTCAGGTTTAGCTGCTACTGGTTTTGCTGCTTCAGGTTTAGCTGCTGGGGGCTGAGGTAATATACCTTTTATTTTATTATCTAATTCTTTAACTTTTTTATCTACTTCTTCTCTTTTACCTTCTTTTGTAGTTTCCCCTTTTTTGATAGCTTC |
|  |  | NJ | GAAGCTATCAAAAAAGGGGAAACTACAAAAGAAGGTAAAAGAGAAGAAGTAGATAAAAAAGTTAAAGAATTAGATAATAAAATAAAAGGTATATTACCTCAGCCCCCAGCAGCTAAACCTGAAGCAGCAAAACCAGTAGCAGCTAAACCTGAAGCAGCAAAACCAGTAGCAGCTAAACCTGAAGCAGCAAAACCAGTAGCAGCTAAACCTGAAGCAGCAAAACCAGTAGCGGCTAAACCTGAAGCAGCTAAACCAGTAGCAGCTAAACCTGAAGCAGCAAAACCAGTAGCAGCTAAACCTGAAGCAGCAAAACCAGTAGCAGCTAAACCTGAAGCAGCTAAACCTGAAGCAGCCAAACCAGTTGCTACTAATACTAATACTAATACTGGCTTTTCACTTACAAATAAACC |
|  |  | XLW-2 | CAAAAAAGGGGAAACTACAAAAGAAGGTAAAAGAGAAGAAGTAGATAAAAAAGTTAAAGAATTAGATAATAAAATAAAAGGTATATTACCTCAGCCCCCAGCAGCTAAACCTGAAGCAGCAAAACCAGTAGCAGCTAAACCTGAAGCAGCAAAACCAGTAGCAGCTAAACCTGAAGCAGCAAAACCAGTAGCAGCTAAACCTGAAGCAGCAAAACCAGTAGCGGCTAAACCTGAAGCAGCTAAACCAGTAGCAGCTAAACCAGTTGCTACTAATACTAATACTAATACTGGCTTTTCACTTACAAATAA |
|  |  | RM48 | GAAGCTATCAAAAAAGGGGAAACTACAAAAGAAGGTAAAAGAGAAGAAGTAGATAAAAAAGTTAAAGAATTAGATAATAAAATAAAAGGTATATTACCTCAGCCCCCAGCAGCTAAACCTGAAGCAGCAAAACCAGTAGCAGCTAAACCTGAAGCAGCAAAACCAGTAGCAGCTAAACCTGAAGCAGCAAAACCAGTAGCAGCTAAACCTGAAGCAGCAAAACCAGTAGCGGCTAAACCTGAAGCAGCTAAACCAGTAGCAGCTAAACCTGAAGCAGCAAAACCAGTAGCAGCTAAACCTGAAGCAGCAAAACCAGTAGCAGCTAAACCTGAAGCAGCTAAACCTGAAGCAGCCAAACCAGTTGCTACTAATACTAATACTAATACTGGCTTTTCACTTACAAATAAACC |
|  |  | LH | TCATTTGAAGCTATCAAAAAAGGGGAAACTACAAAAGAAGGTAAAAGAGAAGAAGTAGATAAAAAAGTTAAGGAATTAGATAATAAAATAAAAGGTATATTACCTCAGCCCCCAGCAGCTAAACCTGAAGCAGCCAAACCAGTAGCGGCTAAACCTGAAGCAGCAAAACCAGTAGCAGCTAAACCTGAAGCAGCAAAACCAGTAGCGGCTAAACCAGAAGCAGCAAAACCAGTAGCGGCTAAACCAGAAGCAGCAAAACCAGTTGCTACTAATACTGGCTTTTCACTTACAAATAAACCAAAAGAAGACTATTT |
|  |  | J | GAAGCTATCAAAAAAGGGGAAACTACAAAAGAAGGTAAAAGAGAAGAAGTAGATAAAAAAGTTAAAGAATTAGATAATAAAATAAAAGGTATATTACCTCAGCCCCCAGCAGCTAAACCTGAAGCGGCTAAACCAGTAGCAGCAAAACCTGAAGCAGCTAAACCTGAAACAACAAAACCAGTAGCAGCTAAACCTGAAGCAGCAAAACCAGTAGCAGCAAAACCAGTAGCAGCAAAACCAGTTGCTACTAATACTAATACTAATACTGGCTTTTCACTTACAAATAAACC |
|  | P97R2 | 168 | AGCGAGTATGAAGAACAAAAAATAATAAAGGAACTAGACAAAACTGTTTTAAATCTTCAATATCAATTCCAGGAAGTCAAGGTAGCTAGTGATCAATATCAGAAACTTAGCCACCCAATGATGACCGAGGGATCTTCAAATCAAGGTAAAAAAGGTGAAGGAACTCCTAATCAAGGTAAAAAAGCAGAGGGTGCTCCTAGTCAAGGTAAAAAAGCCGAAGGAACTTCTAACCAA |
|  |  | 168L | TTTTTACCTAATTCAGGAAGGTAATTAGTTAATTCGGTAGTTGTGTTTTGTTGGTTAGAAGTTCCTTCGGCTTTTTTACCTTGACTAGGAGCACCCTCTGCTTTTTTACCTTGATTAGGAGTTCCTTCACCTTTTTTACCTTGATTTGAAGATCCCTCGGTCATCATTGGGTGGCTAAGTTTCTGATATTGATCACTAGCTACCTTGACTTCCTGGAATTGATATTGAAGATTTAAAACAGTTTTGTCTAGTTCCTTTATTATTTTTTGTTCTTCATACTCGCT |
|  |  | NJ | AGCGAGTATGAAGAACAAAAAATAATAAAGGAACTAGACAAAACTGTTTTAAATCTTCAATATCAATTCCAGGAAGTCAAGGTAGCTAGTGATCAATATCAGAAACTTAGCCACCCAATGATGACCGAGGGATCTTCAAATCAAGGTAAAAAAGGTGAAGGAACTCCTAATCAAGGTAAAAAAGCAGAGGGTGCTCCTAGTCAAGGTAAAAAAGCCGAAGGAACTTCTAACCAACA |
|  |  | XLW-2 | AGCGAGTATGAAGAACAAAAAATAATAAAGGAACTAGACAAAACTGTTTTAAATCTTCAATATCAATTCCAGGAAGTCAAGGTAGCTAGTGATCAATATCAGAAACTTAGCCACCCAATGATGACCGAGGGATCTTCAAATCAAGGTAAAAAAGGTGAAGGAACTCCTAATCAAGGTAAAAAAGCAGAGGGTGCTCCTAGTCAAGGTAAAAAAGCCGAAGGAACTTCTAACCAAC |
|  |  | RM48 | AGCGAGTATGAAGAACAAAAAATAATAAAGGAACTAGACAAAACTGTTTTAAATCTTCAATATCAATTCCAGGAAGTCAAGGTAGCTAGTGATCAATATCAGAAACTTAGCCACCCAATGATGACCGAGGGATCTTCAAATCAAGGTAAAAAAGGTGAAGGAACTCCTAATCAAGGTAAAAAAGCAGAGGGTGCTCCTAGTCAAGGTAAAAAAGCCGAAGGAACTTCTAACCAACAA |
|  |  | LH | AGAACAAGAAATAATAAAGGAACTAGATAAAACTGTTTTAAATCTTCAATATCAATTCCAGGAAGTCAAGGTAACTAGTGACCAATATCAGAAACTTAGCCACCCAATGATGACCGAGGGGACTCAAAACCAAGGTAAAAAAGGTGAAGGAACTCCTAATCAAGGTAAAAAAGCAGAGGGTGCTCCTAGTCAAGGGAAAAAAGCAGAAGGAACTCCTAACCAAGGGAAAAAAGCCGAAGGAACACCTAATCAACAAAGCCCAACTAGCGAATTAACTAATTACCTTCCTGA |
|  |  | J | AGCGAGTATGAAGAACAAAAAATAATAAAGGAACTAGATAAAACTGTTTTAAATCTTCAATATCAATTCCAGGAAGTCAAGGTAACTAGTGAACAATATCAGAAACTTAGCCACCCAATGATGACCGAGGGATCTCCTAATCAAGGTAAAAAAGCCGAAGGCGCTCCTAACCAAGGCAAAAAAGCCGAAGGCGCACCTAGTCAAGGGAAAAAAGCCGAAGGCGCTCCTAACCAAGGCAAAAAAGCCGAAGGCGCACCTAGTCAAGGGAAAAAAGCAGAGGGTGCTTCTAATCAACAAAGCACAACTACCGAATTAACTAA |
|  | P95 | 168 | TTAGCGGAAATGGACTTATTTACAAAGCAAATAGTGTTTTTAAGGACAAATTTGGGAATCTAAAAATTAGATTTGCAGTTAAAGACTTAGATGCAAGTGAGAAAAAACAGATTGTTTTTCCAAATATTTTGGAACCAGAACTAGAAAAAAAAGAACAGGATGTTAGTCCAAAAGGAACAGAAGCTGAAAGTGAACAAGAAAAACAAGAAAAATCTCAATTAGCTTCAGATTCTAGTCAAACTTCAATAACATCTAGCCAAAACCAAGAAAATAATCAAAAAAATCAGCTTGAAATTTTCAAACCCGCAGAAAAGGAAGCAAAATATCCA |
|  |  | 168L | TTTACTTAGCGGAAATGGACTTATTTACAAAGCAAATAGTGTTTTTAAGGACAAATTTGGGAATCTAAAAATTAGATTTGCAGTTAAAGACTTAGATGCAAGTGAGAAAAAACAGATTGTTTTTCCAAATATTTTGGAACCAGAACTAGAAAAAAAAGAACAGGATGTTAGTCCAAAAGGAACAGAAGCTGAAAGTGAACAAGAAAAACAAGAAAAATCTCAATTAGCTTCAGATTCTAGTCAAACTTCAATAACATCTAGCCAAAACCAAGAAAATAATCAAAAAAATCAGCTTGAAATTTTCAAACCCGCAGAAAAGGAAGCAAAATATCCACTTGT |
|  |  | NJ | TACTTAGCGGAAATGGACTTATTTACAAAGCAAATAGTGTTTTTAAGGACAAATTTGGGAATCTAAAAATTAGATTTGCAGTTAAAGACTTAGATGCAAGTGAGAAAAAACAGATTGTTTTTCCAAATATTTTGGAACCAGAACTAGAAAAAAAAGAACAGGATGTTAGTCCAAAAGGAACAGAAGCTGAAAGTGAACAAGAAAAACAAGAAAAATCTCAATTAGCTTCAGATTCTAGTCAAACTTCAATAACATCTAGCCAAAACCAAGAAAATAATCAAAAAAATCAGCTTGAAATTTTCAAACCCGCAGAAAAGGAAGCAAAATATCCACTTGT |
|  |  | XLW-2 | TCCTTTACTTAGCGGAAATGGACTTATTTACAAAGCAAATAGTGTTTTTAAGGACAAATTTGGGAATCTAAAAATTAGATTTGCAGTTAAAGACTTAGATGCAAGTGAGAAAAAACAGATTGTTTTTCCAAATATTTTGGAACCAGAACTAGAAAAAAAAGAACAGGATGTTAGTCCAAAAGCAACAGAAGCTGAAAGTAAACAAGAAAAACAAGAAAAATCTCAATTAGCTTCAGATTCTAGTCAAACTTCAACAACATCTAGCCAAAACCAAGAAAATAATCAAAAAAATCAGCTTGAAATTTTCAAGCCCGCAGAAAAGGAAGCAAAATATCCACTTGT |
|  |  | RM48 | TCCTTTACTTAGCGGAAATGGACTTATTTACAAAGCAAATAGTGTTTTTAAGGACAAATTTGGGAATCTAAAAATTAGATTTGCAGTTAAAGACTTAGATGCAAGTGAGAAAAAACAGATTGTTTTTCCAAATATTTTGGAACCAGAACTAGAAAAAAAAGAACAGGATGTTAGTCCAAAAGGAACAGAAGCTGAAAGTGAACAAGAAAAACAAGAAAAATCTCAATTAGCTTCAGATTCTAGTCAAACTTCAATAACATCTAGCCAAAACCAAGAAAATAATCAAAAAAATCAGCTTGAAATTTTCAAACCCGCAGAAAAGGAAGCAAAATATCCACTTGT |
|  |  | LH | ACTTAGCGGAAATGGACTTATTTACAAAGCAAATAGTGTTTTTAAGGACAAATTTGGGAATCTAAAAATTAGATTTGCAGTTAAAGACTTAGATGCAAGTGAGAAAAAACAGATTGTTTTTCCAAATATTTTAGAACCAGAACTAGAAAAAAAAGAACAGGATGTTAGTCCAAAAGCAACAGAAGCTGAAAGTAAACAAGAAAAACAAGAAAAACAAGAAAAATCTCAATTAGCTTCAGATTCTAGTCAAACTTCAACAACATCTAGCCAAAACCAAGTAAATAATCAAAAAAATCAGCTTGAAATTTTCAAACCTGCAGAAAAAGAAGCAAAATATCC |
|  |  | J | TCCTTTACTTAGCGGAAATGGACTTATTTACAAAGCAAATAGTGTTTTTAAGGACAAATTTGGGAATTTAAAAATTAGAT  TTGCAGTTAAAGACTTAGATGCAAGTGAGAAAAAACAGATTGTTTTTCCAAATATTTTGGAACCAGAACTAGAAAAAAAAGAACAGGATGTTAGTCCAAAAGCAACAGAAGCTGAAAGTAAACAAGAAAAACAAGAAAAATCTCAATTAGCTTCAGATTCTAGTCAAACTTCAATAACATCTAGCCAAAACCAAGAAAATAATCAAAAAAATCAGCTTGAAATTTTCAAACCCGCAGAAAAAGAAGCAAAATATCCACTTGT |
|  | P146R1 | 168 | AGTCACAAAAACCTCAAAGTGAACAAACCGAGATTAAAAAAACTTATTTTGCCGAAATTGATAAAATTTTAAGCAAAAT  AACTATGCGCAAACTTCAGCTTAGCGACTTTAAGGTAGCTCCACAGACAAGTTCTTCGCAACCAAAGCAAGTTAAAGCAA  GTGTGTCAGCTTGATCCAATTTAGATCAAGGGCAAGAAAATTGAATTTTAGTTCCGGTTAGTCAGCAAAGTTTAAATCCA  CAACAACAACAACAACCTCAGCAACCTCAACCTCAGAGTCAGCCTCAAGCTCAAACTCAGCCTAAGGCTCAAACTCAAAG  CTCTCCT |
|  |  | 168L | AGTCACAAAAACCTCAAAGTGAACAAACCGAGATTAAAAAAACTTATTTTGCCGAAATTGATAAAATTTTAAGCAAAAT  AACTATGCGCAAACTTCAGCTTAGCGACTTTAAGGTAGCTCCACAGACAAGTTCTTCGCAACCAAAGCAAGTTAAAGCAA  GTGTGTCAGCTTGATCCAATTTAGATCAAGGGCAAGAAAATAGAATTTTAGTTCCGGTTAGTCAGCAAAGTTTAAATCCA  CAACAACAACAACAACAACCTCAGCAACCTCAACCTCAGAGTCAGCCTCAAGCTCAAACTCAGCCTAAGGCTCAAACTCA  AAGCTCTCCT |
|  |  | NJ | AGTCACAAAAACCTCAAAGTGAACAAACCGAGATTAAAAAAACTTATTTTGCCGAAATTGATAAAATTTTAAGCAAAATAACTATGCGCAAACTTCAGCTTAGCGACTTTAAGGTAGCTCCACAGACAAGTTCTTCGCAACCAAAGCAAGTTAAAGCAAGTGTGTCAGCTTGATCCAATTTAGATCAAGGGCAAGAAAATTGAATTTTAGTTCCGGTTAGTCAGCAAAGTTTAAATCCACAACAACAACAACAACCTCAGCAACCTCAACCTCAGAGTCAGCCTCAAGCTCAAACTCAGCCTAAGGCTCAAACTCAAAGCTCTCC |
|  |  | XLW-2 | AGTCACAAAAACCTCAAAGTGAACAAACCGAGATTAAAAAAACTTATTTTGCCGAAATTGATAAAATTTTAAGCAAAATAACCATGCGCAAACTTCAGCTTAGCGACTTTAAGGTAGCTCCACAGACAAGTTCTTCGCAACCAAAGCAAGTTAAAGCAAGTGTGTCAGCTTGATCCAATTTAGATCAAGGGCAAGAAAATAGAATTTTAGTTCCGGTTAGTCAGCAAAGTTCGAATCCACAACAACAACAACAACAACAACCTCAACCTCAGAGTCAGCCCCAACCTCAACCTCAGAGTCAACCTCAATCTCAGAGTCAGCCTAATGC |
|  |  | RM48 | TAGTCACAAAAACCTCAAAGTGAACAAACCGAGATTAAAAAAACTTATTTTGCCGAAATTGATAAAATTTTAAGCAAAATAACTATGCGCAAACTTCAGCTTAGCGACTTTAAGGTAGCTCCACAGACAAGTTCTTCGCAACCAAAGCAAGTTAAAGCAAGTGTGTCAGCTTGATCCAATTTAGATCAAGGGCAAGAAAATTGAATTTTAGTTCCGGTTAGTCAGCAAAGTTTAAATCCACAACAACAACAACAACAACCTCAGCAACCTCAACCTCAGAGTCAGCCTCAAGCTCAAACTCAGCCTAAGGCTCAAATTCAAAGCTCTC |
|  |  | LH | TAGTCACAAAAACCTCAAAGTGAACAAACCGAGATTAAAAAAACTTATTTTGCCGAAATTGATAAAATTTTAAGCAAAAT  AACCATGCGCAAACTTCAACTTAGCGACTTTAAGGTAGCTCCACAGACAAGTTCTTCGCAACCAAAGCAAGTTAAAGCAA  GTGTGTCAGCTTGATCCAATTTAGATCAAGGGCAAGAAAATAGAATTTTAGTTCCGGTTAGTCAGCAAAGTTCGAATCCA  CAACAACAACAACAACCTCAACCTCAGAGTCAGCCCCAACCTCAACCTCAGAGTCAACCTCAATCTCAGAGTCAGCCTAA  TGCTCAAACTCAGCCTAAAGCTCAAATTCAAAGCTCTCC |
|  |  | J | AGTCACAAAAACCTCAAAGTGAACAAACCGAGATTAAAAAAACTTATTTTGCCGAAATTGATAAAATTTTAAGCAAAATA  ACCATGCGCAAACTTCAGCTTAGCGACTTTAAGGTAGCTCCACAGACAAGTTCTTCGCAACCAAAGCAAGTTAAAGCAAG  TGTGTCAGCTTGATCCAATTTAGATCAAGGGCAAGAAAATAGAATTTTAGTTCCGGTTAGTCAGCAAAGTTCGAATCCACAACAACAACAACAACCTCAACCTCAGAGTCAACCTCAATCTCAGCCGCAGCCTAATGCTCAAACTCAGCCTAAAGCTCAAATTCAAAGCTCTCCTAAAGCTCCAGTCCAAAA |
|  | P146R2 | 168 | AGACCAAAAAGTAGGACATACCCAAAAAGAACTCGATCTAAATCAGAAACTAATTTATCAACTCAGCGAACTACCAGGAACAAGCGCCCAAGGTTCTTCTGGATCTAGTGCACAAACAGAACAAATTAAAGAAGTTAAACTCCCAACACTAACTGCTTTTATTTCAAAACAAGAACTAGAAGCTCTAATTGATGGGGATAGGAATTTAGCTAGTCAGCCAACAAGTCAGGCAGTATCTGTTTCTCAAGAAGTTAAAACAACCGAGTTCCAACAACAAGAGGCAAATTCAACTAATTCTAGCCCAACTAGTCCTAGTCCAAGTCCTAGTCCAAGTTCAGCTAGTCCAACTAGTCCTAAAACGGTAGATGAAAATATAGGGCTACCGAATCCTAGATTTGAGGAAATTAAAAAAATAATTAGTTCCGAGTTTACTTATAAGTATAATTTCCGGGCTAACGAGGCGCTTTTAGATGCCTGAGTT |
|  |  | 168L | AGACCAAAAAGTAGGACATACCCAAAAAGAACTCGATCTAAATCAGAAACTAATTTATCAACTCAGTGAACTACCAGGAACAAGCGCCCAAGGTTCTTCTGGATCTAGTGCACAAACAGAACAAATTAAAGAAGTTAAACTCCCAACACTAACTGCTTTTATTTCAAAACAAGAACTAGAAGCTCTAATTGATGGGGATAGGAATTTAGCTAGTCAGCCAACAAGTCAGGCAGTATCTGTTTCTCAAGAAGTTAAAACAACCGAGTTCCAACAACAAGAGGCAAATTCAACTAATTCTAGCCCAACTAGTCCTAGTCCAAGTCCTAGTCCAAGTTCAGCTAGTCCAACTAGTCCTAAAACGGTAGATGAAAATATAGGGCTACCGAATCCTAGATTTGAGGAAATTAAAAAAATAATTAGTTCCGAGTTTACTTATAAGTATAATTTCCGGGCTAACGAGGCGCTTTTAGATGCCTGAGTT |
|  |  | NJ | AGACCAAAAAGTAGGACATACCCAAAAAGAACTCGATCTAAATCAGAAACTAATTTATCAACTCAGTGAACTACCAGGAACAAGCGCCCAAGGTTCTTCTGGATCTAGTGCACAAACAGAACAAATTAAAGAAGTTAAACTCCCAACACTAACTGCTTTTATTTCAAAACAAGAACTAGAAGCTCTAATTGATGGGGATAGGAATTTAGCTAGTCAGCCAACAAGTCAGGCAGTATCTGTTTCTCAAGAAGTTAAAACAACCGAGTTCCAACAACAAGAGGCAAATTCAACTAATTCTAGCCCAACTAGTCCTAGTCCAAGTCCTAGTCCAAGTTCAGCTAGTCCAACTAGTCCTAAAACGGTAGATGAAAATATAGGGCTACCGAATCCTAGATTTGAGGAAATTAAAAAAATAATTAGTTCCGAGTTTACTTATAAGTATAATTTCCGGGCTAACGAGGCACTTTTAGATGCTTGAGTT |
|  |  | XLW-2 | TAAGACCAAAAAGTAGGACATACCCAAAAAGAACTCGATCTAAATCAGAAACTAATTTATCAACTCAGTGAACTACCAGGAACTAGCACCCAAGCTTCTTCTGGATCTAGTCCGCAAGCAGAACAAATTAAAGAAGTTAAACTCCCAACACTAACTGCTTTTATTTCAAAACAAGAACTAGAAGCTCTAATTGATGGGGATAAGAATTTAGCTAGTCAGCCAACAAGTCAGGCAATATCTGTTTCTCAAGAAGCTAAAACAACCGAGTTCCAACAGCAAGAGGCAAATTCAACTAATTCTAGTCCAACTAGTCCAAGCCCAAGCCCTAGTCCAAGTAGCCCTAGTCCAAGTCCAGCTAGTCCAACTAGTCCTAAAAATGTCGATGAAAATATAGGAGTGCCAAATCCTAGATTTGAGGAAATTAAAAAAATAATTAGTTCCGAGTTTACTTATAAGTATAATTTTCGGGCTAACGAGGCACTTTTAGATGCTTGAGTT |
|  |  | RM48 | AGACCAAAAAGTAGGACATACCCAAAAAGAACTCGATCTAAATCAGAAACTAATTTATCAACTCAGTGAACTACCAGGAACAAGCGCCCAAGGTTCTTCTGGATCTAGTGCACAAACAGAACAAATTAAAGAAGTTAAACTCCCAACACTAACTGCTTTTATTTCAAAACAAGAACTAGAAGCTCTAATTGATGGGGATAGGAATTTAGCTAGTCAGCCAACAAGTCAGGCAGTATCTGTTTCTCAAGAAGTTAAAACAACCGAGTTCCAACAACAAGAGGCAAATTCAACTAATTCTAGCCCAACTAGTCCTAGTCCAAGTCCTAGTCCAAGTTCAGCTAGTCCAACTAGTCCTAAAACGGTAGATGAAAATATAGGGCTACCGAATCCTAGATTTGAGGAAATTAAAAAAATAATTAGTTCCGAGTTTACTTATAAGTATAATTTCCGGGCTAACGAGGCACTTTTAGATGCTTGAGTTAGG |
|  |  | LH | TAAGACCAAAAAGTAGGACATACCCAAAAAGAACTCGATCTAAATCAGAAACTAGTTTATCAACTCAGTGAACTACCAGGAACTAGCACCCAAGGTTCTTCTGGATCTAGTACACAAACCGAACAAATTAAAGAAGTTAAACTCCCAACACTAACTGCTTTTATTTCAAAACAAGAACTAGAAGCTCTAATTGATGGGGATAAGAATTTAGCTAGTCAGCCAACAAGTCAAGCAGTATCTGTTTCTCAAGTTAAAGCAACGGAGTTCCAACAGCAAGAGGCAAATTCAACTAATTCTAGTCCAACTAGTCCAAGCCCTAGTCCAACTAGTCCAAGCCCAGCTAGTCCAAGTTCAAGCCCTAGTCCAACTAGTCCTAAAAATCTCGATGAAAATATAGGAGTGCCAAATCCTAGATTTGAGGAAATTAAAAAAATAATTAGTTCCGAGTTTACTTATAAGTATAATTTTCGTGCTAACGAGGCACTTTTA |
|  |  | J | AGACCAAAAAGTAGGACATACCCAAAAAGAACTCGATCTAAATCAGAAACTAGTTTATCAACTCAGTGAACTACCAGGAACTAGCACCCAAGGTTCTTCTGGATCTAGTACACAAACCGAACAAATTAAAGAAGTTAAACTCCCAACACTAACTGCTTTTATTTCAAAACAAGAACTAGAAGCTCTAATTGATGGGGATAAGAATTTAGCTAGTCAGCCAACAAGTCAAGCAGTATCTGTTTCTCAAGTTAAAGCAACGGAGTTCCAACAGCAAGACGCAAATTCAACTAATTCTAGTCCAACTAGTCCAAGCCCTAGTCCAACTAGTCCAAGTCCAGCTAGTCCAAGTTCAAGTCCTAGTCCAACTAGTCCTAAAAATCTCGATGAAAATATAGGAGTGCCAAATCCTAGATTTGAGGAAATTAAAAAAATAATTAGTTCCGAGTTTACTTATAAGTATAATTTTCGTGCTAACGAGGCACTTTTAGATGCTTGAGTT |
|  | P146R3 | 168 | CCAAAGTAGTGATTCAAGTGATTCAAAATCAGATTCAAGCGATTCTTCAGATTCTAAGACTACTGCTGCAAATCAAGA  TCTTCAAAGTAAATTAACTAACCTTAAATCTCAAATAGAGGCTATAGTTAAAAAATATGAAAGTGAGTCTAAAAAGTATT  TAGGGACCGAAAATGGTAATGGGGGGAGCAGCTCAAGCGGAGAGCAGAAAGGCTCATCTATCCCTGAAGAAAATAAAAAA  TTCATCTTGGAGAATACAGCAAAACTTGATAATTTAGCCGATCTACTTTTAGCTTTCTATTATCAGGCTAAAAGATTAAA  TTTTGCAAGTTGAAGTCAACTCCAAGACGAAGATCTTGACTATCAAATACAATTTGAAAAAGAGGCTAATACCACTGAGT  CTTCTTCATCTTCTTCATCTTCCTCACCCTCTTCTTCTGAAACCGATACAA |
|  |  | 168L | CCAAAGTAGTGATTCAAGTGATTCAAAATCAGATTCAAGCGATTCTTCAGATTCTAAGACTACTGCTGCAAATCAAGA  TCTTCAAAGTAAATTAACTAACCTTAAATCTCAAATAGAGGCTATAGTTAAAAAATATGAAAGTGAGTCTAAAAAGTATT  TAGGGACCGAAAATGGTAATGGGGGGAGCAGCTCAAGCGGAGAGCAGAAAGGCTCATCTATCCCTGAAGAAAATAAAAAA  TTCATCTTGGAGAATACAGCAAAACTTGATAATTTAGCCGATCTACTTTTAGCTTTCTATTATCAGGCTAAAAGATTAAA  TTTTGCAAGTTGAAGTCAACTCCAAGACGAAGATCTTGACTATCAAATACAATTTGAAAAAGAGGCTAATACCACTGAGT  CTTCTTCATCTTCTTCATCTTCCTCACCCTCTTCTTCTGAAACCGATACAA |
|  |  | NJ | CCAAAGTAGTGATTCAAGTGATTCAAAATCAGATTCAAGCGATTCTTCAGATTCTAAGACTACTGCTGCAAATCAAGATCTTCAAAGTAAATTAACTAACCTTAAATCTCAAATAGAGGCTATAGTTAAAAAATATGAAAGTGAGTCTAAAAAGTATTTAGGGACCGAAAATGGTAATGGGGGGAGCAGCTCAAGCGGAGAGCAGAAAGGCTCATCTATCCCTGAAGAAAATAAAAAATTCATCTTGGAGAATACAGCAAAACTTGATAATTTAGCCGATCTACTTTTAGCTTTCTATTATCAGGCTAAAAGATTAAATTTTGCAAGTTGAAGTCAACTCCAAGACGAAGATCTTGACTATCAAATACAATTTGAAAAAGAGGCTAATACCACTGAGTCTTCTTCATCTTCTTCATCTTCCTCACCCTCTTCTTCTGAAACCGATACAA |
|  |  | XLW-2 | ACCCAAAGTAGTGATTCAAGTGATTCAAAATCAGATTCAAGCGATTCTTCAGATTCTAAGACCACTTCTACAAAGCAAGATCTTCTAAGTAAATTAACTAGCCTTAAATCTCAAATAGAGGCTATAGTTAAAAAATATGAAACAGAGTCTAAAAATTATTTAGGGACCCAAAATAATAATGGTGGCAGCAGCTCAGGTACAGAACAGAAGGGCTCATCTATCCCTGAAGAAAATAAAAAATTCATCTTGGAAAATACAGCAAAACTTGATAATTTAGCCGATCTACTTTTAGCTTTCTATTATCAGGCTAAAAGATTAAATTTTGCAAGTTGAAGTCAACTCCAAGACGAAGATCTTGACTATCAAATACAATTTGAGAAAGAGGCTAATAACACTGAGTCTTCTTCATCCTCTTCATCTTCATCTTCTTCTTCTTCTTCTTCTTCTTCATCTTCTTCTGAAACCGATACA |
|  |  | RM48 | AGTAGTGATTCAAGTGATTCAAAATCAGATTCAAGCGATTCTTCAGATTCTAAGACTACTGCTGCAAATCAAGATCTTCAAAGTAAATTAACTAACCTTAAATCTCAAATAGAGGCTATAGTTAAAAAATATGAAAGTGAGTCTAAAAAGTATTTAGGGACCGAAAATGGTAATGGGGGGAGCAGCTCAAGCGGAGAGCAGAAAGGCTCATCTATCCCTGAAGAAAATAAAAAATTCATCTTGGAGAATACAGCAAAACTTGATAATTTAGCCGATCTACTTTTAGCTTTCTATTATCAGGCTAAAAGATTAAATTTTGCAAGTTGAAGTCAACTCCAAGACGAAGATCTTGACTATCAAATACAATTTGAAAAAGAGGCTAATACCACTGAGTCTTCTTCATCTTCTTCATCTTCCTCACCCTCTTCTTCTGAAACCGATACAA |
|  |  | LH | ACCCAAAGTAGTGATTCAAGTGATTCAAAATCAGATTCAAGTGATTCTTCAGATGCTAAGACCACTTCTACAAAGCAAGA  TCTTCTAAGTAAATTAACTAGCCTTAAATCTCAAATAGAGGCTATAGTTAAAAAATATGAAACAGAGTCTAAAAATTATT  TAGGGACCCAAAATAATAATGGTGGCAACAGCTCAGGTACAGAACAGAAGGGCTCATCTATCCCTGAAGAAAATAAAAAA  TTCATCTTGGAAAATACAGCAAAACTTGATAATTTAGCCGATCTACTTTTAGCTTTCTATTACCAGGCTAAAAGATTAAA  TTTTGCAAGTTGAAGTCAACTCCAAGACGAAGATCTTGACTATCAAATACAATTTGAGAAAGAGGCTAATAACACTGAGT  CTTCATCCTCTTCATCCTCTTCATCTTCTTCATCCTCTTCATCTTCATCTTCTTCTTCTTCATCTTCTTCTGAACCCGAT  ACA |
|  |  | J | ACCCAAAGTAGTGATTCAAGTGATTCAAAATCAGATTCAAGTGATTCTTCAGATGCTAAGACCACTTCTACAAAGCAAGATCTTCTAAGTAAATTAACTAGCCTTAAATCTCAAATAGAGGCTATAGTTAAAAAATATGAAACAGAGTCTAAAAAGTATTTAGGGACCGAAAATAATAATGGTGGCAGCAGCTCAGGTACAGAACAGAAGGGCTCATCTATCCCTGAAGAAAATAAAAAATTCATCTTGGAAAATACAGCAAAACTTGATAATTTAGCCGATCTACTTTTAGCTTTCTATTATCAGGCTAAAAGATTAAATTTTGCAAGTTGAAGTCAACTCCAAGACGAAGATCTTGACTATCAAATACAATTTGAGAAAGAGGCTAATAACACTGAGTCTTCATCCTCTTCATCCTCTTCATCTTCATCTTCTTCTTCTTCATCTTCTTCTGAAA |
|  | P216R1 | 168 | AATGTAAATGTCTCAACTTTTGGTTCAATAATCGAGTCCCCTTATTTTAGTACTAATTTCCAAGAAGAATCTGACTTAGA  CCAAGAAGGACAAGATGATTCAAAACAAGGAAATAATAGCCTAGATAATCAAGAAGCAGGTCTTTTAAAACAAAAACTGG  CAATTTTATTAGGGAATCAATTTATCCAATATTATCAACAAAATGATAAGGAAATTGAATTCGAGATTATCAATGTTGAG  AAAGTTTCAGAGCTTAGTTTCCGCGTTGAATTTAAATTAGCAAAAACTCTTGAAGACAACGGAAAAACTATTCGAGTTTT  ATCTGATGAGACAATGTCATTAATTGTTAATACTACAATTGAAAAAGCACCAGAAATGAGTGCGGTTCCCGAAGTATTTG  ATACTAAATGGGTTGAGCAATATGATCCAAGAACCCCGCTTGCGGCTAAGACAAAGTTTGTCTTAAAATTCAAAGATCAA  ATACCAGTTGATGGCAGCGGAAATATTTCTGATAAATGACTAGCAAGTATTCCTTTGGTGATTCACCAGCAAATGTTGCG  TCTTAGCCCTGTAGTTAAAACGATAAGAGAGCTCGGTCTAAAGACCGAACAACAACAACAACAACAACAAAAGAAAGCTG  TTAGAAAAGAA |
|  |  | 168L | AATGTAAATGTCTCAACTTTTGGTTCAATAATCGAGTCCCCTTATTTTAGTACTAATTTCCAAGAAGAATCTGACTTAGA  CCAAGAAGGACAAGATGATTCAAAACAAGGAAATAATAGCCTAGATAATCAAGAAGCAGGTCTTTTAAAACAAAAACTGG  CAATTTTATTAGGGAATCAATTTATCCAATATTATCAACAAAATGATAAGGAAATTGAATTCGAGATTATCAATGTTGAG  AAAGTTTCAGAGCTTAGTTTCCGCGTTGAATTTAAATTAGCAAAAACTCTTGAAGACAACGGAAAAACTATTCGAGTTTT  ATCTGATGAGACAATGTCATTAATTGTTAATACTACAATTGAAAAAGCACCAGAAATGAGTGCGGTTCCCGAAGTATTTG  ATACTAAATGGGTTGAGCAATATGATCCAAGAACCCCGCTTGCGGCTAAGACAAAGTTTGTCTTAAAATTCAAAGATCAA  ATACCAGTTGATGGCAGCGGAAATATTTCTGATAAATGACTAGCAAGTATTCCTTTGGTGATTCACCAGCAAATGTTGCG  TCTTAGCCCTGTAGTTAAAACGATAAGAGAGCTCGGTCTAAAGACCGAACAACAACAACAACAACAACAAAAGAAAGCTG  TTAGAAAAGAA |
|  |  | NJ | AATGTAAATGTCTCAACTTTTGGTTCAATAATCGAGTCCCCTTATTTTAGTACTAATTTCCAAGAAGAATCTGACTTAGACCAAGAAGGACAAGATGATTCAAAACAAGGAAATAATAGCCTAGATAATCAAGAAGCAGGTCTTTTAAAACAAAAACTGGCAATTTTATTAGGGAATCAATTTATCCAATATTATCAACAAAATGATAAGGAAATTGAATTCGAGATTATCAATGTTGAGAAAGTTTCAGAGCTTAGTTTCCGCGTTGAATTTAAATTAGCAAAAACTCTTGAAGACAACGGAAAAACTATTCGAGTTTTATCTGATGAGACAATGTCATTAATTGTTAATACTACAATTGAAAAAGCACCAGAAATGAGTGCGGTTCCCGAAGTATTTGATACTAAATGGGTTGAGCAATATGATCCAAGAACCCCGCTTGCGGCTAAGACAAAGTTTGTCTTAAAATTCAAAGATCAAATACCAGTTGATGGCAGCGGAAATATTTCTGATAAATGACTAGCAAGTATTCCTTTGGTGATTCACCAGCAAATGTTGCGTCTTAGCCCTGTAGTTAAAACGATAAGAGAGCTCGGTCTAAAGACCGAACAACAACAACAACAACAACAAAAGAAAGCTGTTAGAAAAGAAGAAGAACT |
|  |  | XLW-2 | AATGTAAATGTCTCAACTTTTGGTTCAATAATCGAGTCCCCTTATTTTAGTACTAATTTCCAAGAAGAATCTGACTTAGACCAAGAAGGACAAGATGATTCAAAACAAGGAAATAATAGCCTAGATAATCAAGAAGCAGGTCTTTTAAAACAAAAACTGGCAATTTTATTAGGGAATCAATTTATCCAATATTATCAACAAAATGATAAGGAAATTGAATTCGAGATTATCAATGTTGAGAAAGTTTCAGAGCTTAGTTTCCGCGTTGAATTTAAATTAGCAAAAACTCTTGAAGACAACGGAAAAACTATTCGAGTTTTATCTGATGAGACAATGTCATTAATTGTTAATACTACAATTGAAAAAGCACCAGAAATGAGTGCGGTTCCCGAAGTATTTGATACTAAATGGGTTGAGCAATATGATCCAAGAACCCCGCTTGCGGCTAAGACAAAGTTTGTCTTAAAATTCAAAGATCAAATACCAGTTGATGGCAGCGGAAATATTTCTGATAAATGACTAGCAAGTATTCCTTTGGTGATTCACCAGCAAATGTTGCGTCTTAGCCCTGTAGTTAAAACGATAAGAGAGCTCGGTCTAAAGACCGAACAACAACAACAACAACAACAACAAAAGAAAGCTGTTAGAAAAGAAGAAGAACTGGA |
|  |  | RM48 | AAATGTAAATGTCTCAACTTTTGGTTCAATAATCGAGTCCCCTTATTTTAGTACTAATTTCCAAGAAGAATCTGACTTAGACCAAGAAGGACAAGATGATTCAAAACAAGGAAATAATAGCCTAGATAATCAAGAAGCAGGTCTTTTAAAACAAAAACTGGCAATTTTATTAGGGAATCAATTTATCCAATATTATCAACAAAATGATAAGGAAATTGAATTCGAGATTATCAATGTTGAGAAAGTTTCAGAGCTTAGTTTCCGCGTTGAATTTAAATTAGCAAAAACTCTTGAAGACAACGGAAAAACTATTCGAGTTTTATCTGATGAGACAATGTCATTAATTGTTAATACTACAATTGAAAAAGCACCAGAAATGAGTGCGGTTCCCGAAGTATTTGATACTAAATGGGTTGAGCAATATGATCCAAGAACCCCGCTTGCGGCTAAGACAAAGTTTGTCTTAAAATTCAAAGATCAAATACCAGTTGATGGCAGCGGAAATATTTCTGATAAATGACTAGCAAGTATTCCTTTGGTGATTCACCAGCAAATGTTGCGTCTTAGCCCTGTAGTTAAAACGATAAGAGAGCTCGGTCTAAAGACCGAACAACAACAACAACAAAAGAAAGCTGTTAGAAAAGAAGAAAGAACTGGA |
|  |  | LH | ATGTAAATGTCTCAATTTTTGGTTCAATAATCGAGTCCCCTTATTTTAGTACTAATTTCCAAGAAGAAGCTGATTTAGACCAAGAAGGACAAGATGATTCAAAACAAGGAAATAAGAGCCTAGATAATCAAGAAGCAGGTCTTTTAAAACAAAAACTGGCAATTTTATTAGGGAATCAATTTATCCAATATTATCAACAAAATGATAAAGAAATTGAATTCGAGATTATCAATGTTGAGAAAGTTTCAGAGCTTAGTTTCCGCGTTGAATTTAAATTAGCAAAAACTCTTGAAGACAACGGAAAAACTATTCGAGTTTTATCAGATGAGACAATGTCATTAATTGTTAATACTACAATTGAAAAAGCACCAGAAATGAGTGCTGCTCCCGAAGTATTCGATACTAAATGGGTTGAGCAATATGATCCAAGAACCCCGCTTGCGGCTAAGACAAAGTTTGTCTTAAAATTCAAAGATCAAATACCAGTGGATGGCAGCGGAAATATTTCTGATAAATGACTAGCAAGTATTCCTTTGGTGATTCACCAGCAAATGTTGCGTCTTAGCCCGGTAGTTAAAACGATAAGAGAGCTCGGTCTAAAGACCGAACAACAACAACAACAACAACAACAACAACAACAAAAGAAAGCTGTTAGAAAAGAAG |
|  |  | J | AAATGTAAATGTCTCAACTTTTGGTTCAATAATCGAGTCCCCTTATTTTAGTACTAATTTCCAAGAAGAAGCTGATTTAGACCAAGAAGGACAAGATGATTCAAAACAAGGAAATAAGAGCCTAGATAATCAAGAAGCAGGTCTTTTAAAACAAAAACTGGCAATTTTATTAGGGAATCAATTTATCCAATATTATCAACAAAATGATAAAGAAATTGAATTCGAGATTATCAATGTTGAGAAAGTTTCAGAGCTTAGTTTCCGCGTTGAATTTAAATTAGCAAAAACTCTTGAAGACAACGGAAAAACTATTCGAGTTTTATCAGATGAGACAATGTCATTAATTGTTAATACTACAATTGAAAAAGCACCAGAAATGAGTGCTGCTCCCGAAGTATTCGATACTAAATGGGTTGAGCAATATGATCCAAGAACCCCGCTTGCGGCTAAGACAAAGTTTGTCTTAAAATTCAAAGATCAAATACCAGTTGATGCCAGCGGAAATATTTCTGATAAATGACTAGCAAGTATTCCTTTGGTGATTCACCAGCAAATGTTGCGTCTTAGCCCGGTAGTTAAAACAATAAGAGAGCTTGGTCTAAAAACTGAACAACAACAACAACAACAACAACAACAACAAAAGAAAGCTGTTAGAAAAGAAGAAGAACTG |
