# Supplementary material for: Genotyping and biofilm formation of Mycoplasma hyopneumoniae and their association with virulence
Source: Vet Res. 2022 Nov 17;53:95. doi: 10.1186/s13567-022-01109-x (PMC9673451; doi:10.1186/s13567-022-01109-x)
Supplement: Supplementary file 5 — Additional file 5. Correlation of virulence and biofilm. [file 13567_2022_1109_MOESM5_ESM.doc]

**Additional file 5. Correlation of virulence and biofilm.**

The microtiter plate biofilm assay (OD570nm) and R/G ratio were examined by single Sample K-S test. Both variables OD570nm and R/G ratio were normally distributed (*p* > 0.05). Thus, Pearson’s correlation coefficient was calculated. Data were analyzed using SPSS version 18 (SPSS Inc,2009, Chicago, IL, USA) .

Additional file 5A. The average value of OD570 and R/G ratio

| **Strains** | **OD_570_** | **R/G ratio** |
| --- | --- | --- |
| LH2010 | 0.62 | 1.846667 |
| NJ | 0.5733333 | 1.766667 |
| 168 | 0.58 | 1.75 |
| J | 0.3466667 | 1.463333 |
| 168L | 0.11 | 1.186667 |
| XLW-2 | 0.1033333 | 1.15 |
| RM48 | 0.14 | 1.06 |
| Control | 0.0203333 | 0.9 |

Additional file 5B. The results of the K-S test for OD570nm and R/G ratio

|  | **OD570nm** | **R/G ratio** |
| --- | --- | --- |
| N | 8 | 8 |
| Mean±Std.Deviation | 0.3117±0.24934 | 1.3904±0.36515 |
| Kolmogorov-Smirnov Z | 0.254 | 0.213 |
| Asymp. Sig.(2-tailed) | 0.136 | 0.200 |
